# Metagenomic sequencing of marine periphyton: taxonomic and functional insights into biofilm communities

**DOI:** 10.3389/fmicb.2015.01192

**Published:** 2015-10-30

**Authors:** Kemal Sanli, Johan Bengtsson-Palme, R. Henrik Nilsson, Erik Kristiansson, Magnus Alm Rosenblad, Hans Blanck, Karl M. Eriksson

**Affiliations:** ^1^Department of Biological and Environmental Sciences, University of GothenburgGothenburg, Sweden; ^2^Department of Infectious Diseases, Institute of Biomedicine, The Sahlgrenska Academy, University of GothenburgGothenburg, Sweden; ^3^Department of Mathematical Sciences, Chalmers University of TechnologyGothenburg, Sweden; ^4^Department of Chemistry and Molecular Biology, University of GothenburgGothenburg, Sweden; ^5^Department of Shipping and Marine Technology, Chalmers University of TechnologyGothenburg, Sweden

**Keywords:** shotgun metagenomics, microbial ecology, marine biofilms, biofouling, next generation sequencing, shotgun sequencing, biodiversity, pathway analysis

## Abstract

Periphyton communities are complex phototrophic, multispecies biofilms that develop on surfaces in aquatic environments. These communities harbor a large diversity of organisms comprising viruses, bacteria, algae, fungi, protozoans, and metazoans. However, thus far the total biodiversity of periphyton has not been described. In this study, we use metagenomics to characterize periphyton communities from the marine environment of the Swedish west coast. Although we found approximately ten times more eukaryotic rRNA marker gene sequences compared to prokaryotic, the whole metagenome-based similarity searches showed that bacteria constitute the most abundant phyla in these biofilms. We show that marine periphyton encompass a range of heterotrophic and phototrophic organisms. Heterotrophic bacteria, including the majority of proteobacterial clades and *Bacteroidetes*, and eukaryotic macro-invertebrates were found to dominate periphyton. The phototrophic groups comprise *Cyanobacteria* and the alpha-proteobacterial genus *Roseobacter*, followed by different micro- and macro-algae. We also assess the metabolic pathways that predispose these communities to an attached lifestyle. Functional indicators of the biofilm form of life in periphyton involve genes coding for enzymes that catalyze the production and degradation of extracellular polymeric substances, mainly in the form of complex sugars such as starch and glycogen-like meshes together with chitin. Genes for 278 different transporter proteins were detected in the metagenome, constituting the most abundant protein complexes. Finally, genes encoding enzymes that participate in anaerobic pathways, such as denitrification and methanogenesis, were detected suggesting the presence of anaerobic or low-oxygen micro-zones within the biofilms.

## Introduction

Metagenomics is defined as the culture-independent genomic analysis of an assemblage of microorganisms (Riesenfeld et al., 2004) and has had a profound impact on our understanding of the biosphere (O’Malley and Dupré, 2009; Heidelberg et al., 2010; Tseng and Tang, 2014). The majority of published metagenomics studies so far has focused either on free-living microbiota such as plankton in the aquatic environment, microbes in air and soil, or host-associated communities living in and on plants and animals. The biofilm form of microbial life, on the other hand, is less frequently sampled, and a search (15th of November 2014) for the term “biofilm” in the available metagenome data in the MG-RAST database (Glass et al., 2010), revealed that less than 5% stem from microbial biofilms.

Biofilms are highly structured agglomerations of micro organisms that are attached to surfaces and are ubiquitous in the environment (Costerton et al., 1995). Periphyton communities are the phototrophic, multi-species biofilms found on submerged substrata in aquatic environments (Wetzel, 1975) and harbors viruses, bacteria, algae, fungi, and proto- and metazoans. The biofilm structure is held together by a matrix of EPSs including polysaccharides, proteins, nucleic acids, phospholipids, and humic substances (Flemming and Wingender, 2010). The EPS matrix confers adhesive properties to the biofilm for surface attachment and mechanical stability. Furthermore, it functions as an external compartment for organisms where degradation of xenobiotics and biomolecules (Wuertz et al., 2004), intercellular communication (de Kievit, 2009), horizontal gene transfer (Chia et al., 2008), and various other collaborative and antagonistic interactions take place. The heterogeneity of biofilms and the metabolism of the various biofilm members, results in physicochemical micro-zones with sharp gradients of metabolic substrates and products (Stewart and Franklin, 2008).

Analysis of species composition in periphyton communities has a long history in the field of monitoring river water quality. Since anthropogenic and natural perturbations are known to induce alterations in community structure, periphyton can exhibit early signs of changes in the environment (Sabater et al., 2007; Schneider and Lindstrom, 2011). Researchers have used the bioindicator potential of periphyton for monitoring of environmental status (Tilley and Haushild, 1975; Smucker and Vis, 2009) and for risk assessment of chemicals (Geiszinger et al., 2009). Marine periphyton is also relevant for a problem of high economic impact on the maritime industry, namely biofouling, which is the undesirable accumulation of biota on underwater surfaces, such as ship hulls. Periphyton accommodates the larvae of, e.g., barnacles and mussels that later develop into adults on ship hulls and severely increase the drag force, the fuel consumption, and the exhaust emissions from ships (Abbott et al., 2000).

Periphyton is composed of a large number of species from different levels of the food web. Partial understanding of the periphytic diversity has been obtained either by laborious microscopy examinations or by laboratory culture isolates. Although photosynthetic pigment analysis and polymerase chain reaction (PCR)-based molecular identification techniques have provided agile solutions for the evaluation of periphytic community structure, they both describe a subset of the total diversity due to their technical restrictions. For example, Eriksson et al. (2009a,b) used light microscopy and PCR amplified clone libraries of the *psbA* gene and described a large diversity of phototrophic organisms in marine periphyton from the Swedish west coast. Also, Porsbring et al. (2007) used light microscopy and HPLC-based analysis of pigment profiles of marine periphyton from the same area, and showed that changes in composition of phototrophic species was reflected in pigment profiles. Still, the restrictions of these techniques have left the scientific community with a limited view of the total diversity and functionality present in periphyton communities. The use of shotgun metagenomic sequencing can improve our knowledge about periphyton community structure and functioning since it provides an efficient culture-independent method by enabling direct identification of genetic material from environmental samples.

In this study, we used shotgun metagenomic sequencing to characterize the community structure of periphyton from the marine environment of the Swedish west coast. We analyzed metagenomes from five sites in the coastal archipelago as well as one metagenome with pooled DNA from four sampling occasions at the fifth site. This sampling strategy was used to get a broader view on periphytic diversity. Due to the limited knowledge about organisms in marine periphyton communities we here aim to describe the variability in community structure and of genes encoding community functions in the sampled area. We also assess the genes encoding proteins in the metabolic pathways that predispose periphytic organisms for their attached lifestyle. We finally make an attempt at examining the contribution of different taxonomic groups to some of the different energy metabolism pathways in these biofilm communities.

## Materials and Methods

### Sample Collection

Periphyton communities were allowed to colonize and grow on rectangular glass slides (150 mm × 20 mm) at 1.5 m depth at five sampling sites at the mouth area of the Gullmar fjord on the Swedish west coast. Five sites were sampled in order to get a broader overview of periphyton composition in this area. The samples represent five relatively nearby sites (the two most distant sites are 11 km apart) from the inner to the outer archipelago of the Swedish west coast, as well as an integrated sample of four sampling times from late April to late September in the outer archipelago (**Table 1**). For each site one sample, corresponding to a surface area of approximately 100 cm^2^, was taken. Although the sites are different in terms of water depth, bottom characteristics and distance to land, they are connected by water movement from currents, tide and weather-dependent high and low waters, and by the associated drifting cells and organisms. Samples 1–4 and 5a were sampled from the five sites on 23rd of July 2004. Sample 5b was obtained by pooling equal amounts of DNA from four different sampling occasions (28th of April, 23rd of July, 30th of August and 21st of September) at site 5. All samples were independent and were sampled after 2.5 weeks of colonization and growth. Physico-chemical parameters of the sea water in this region is given in Supplementary Table S1. The slides were transported to the lab in seawater collected at the site and protected from strong sunlight and temperature changes. The communities were sampled by scraping off the biota from the slides with a sterile scalpel into sterile filtered seawater (Merck Millipore, Darmstadt, Germany). Samples were then centrifuged at 6,500 *g* for 10 min and the resulting pellets were snap-frozen in liquid nitrogen and stored at -80°C. DNA extraction was done according to the Plant DNAzol reagent protocol (Life Technologies, Stockholm, Sweden). Isolated DNA from the samples was multiplexed and sequenced in a Roche 454 GS-FLX system using Titanium chemistry at the Max Planck Institute for Molecular Genetics in Berlin, Germany. Descriptions of the sampling sites, along with information on the sequence reads from each site, are given in **Table 1**.

**Table 1:** Sampling information.

Sample	Coordinates (latitude, longitude)	Water depth (m)	Description	Number of sequences before trimming	Number of sequences after trimming	Mean sequence length (bp)	Number of predicted genes	GC content (%)
1	58.24326, 11.46282	4	Inner part of muddy bay.	143143	94945	338 ± 102	95690	42.85
2	58.25143, 11.46059	6	Mouth of the muddy bay of site 1.	157401	102621	344 ± 101	103870	41.79
3	58.25738, 11.46346	10	Lee side of small rocky shore islet.	132441	88507	333 ± 102	86566	38.01
4	58.23136, 11.39997	4	Muddy bay of an island in the outer archipelago.	162492	101750	348 ± 100	106171	46.77
5a	58.21025, 11.31431	6	Lee side of outmost islet in the archipelago.	132079	86030	335 ± 100	79970	35.76
5b	Same as 5a.	Same as 5a.	Same as 5a.	117914	76817	343 ± 101	77423	41.74
Total				845470	550670	340 ± 101	549690	

*Description of sites and basic sequencing statistics of periphyton metagenome samples.*

### Bioinformatics Analysis

#### Sequence Processing

Raw sequencing data with a maximum of two erroneous bases in the barcode region were used for the sequence analysis. The sff-extract script (http://bioinf.comav.upv.es/sff_extract/) was run to convert standard flowgram format (SFF) data to FASTA-formatted sequence reads along with their read quality scores. The script options were set to remove the barcode and adaptor regions from each read. Duplicate sequences were eliminated from the raw reads by using cd-hit-454 (Niu et al., 2010). The sequence reads were further quality-trimmed by an average quality threshold score of Q20 per fixed window size of 50 bases (Schloss et al., 2009). This operation kept 65% of the original reads, and all the taxonomic and functional annotation and analyses were based on these quality-filtered sequence reads. The total number of sequences extracted and the total number of sequences after quality trimming as well as the average sequence length and average GC content of each sample are listed in **Table 1**. The trimmed sequence datasets are deposited in, and available for download from, MG-RAST (Glass et al., 2010) with the identifiers 4508981.3, 4508982.3, 4508983.3, 4508984.3, 4508985.3, and 4508986.3 for samples 1, 2, 3, 4, 5a,b, respectively.

#### Taxonomy Assignment and Analysis

The taxonomic diversity was assessed using both marker genes and whole metagenome similarity searches. The marker gene approach used the ribosomal (SSU/16S/18S) gene as basis. Metaxa v 1.02 (Bengtsson et al., 2011) was used to extract SSU rRNA sequences from the metagenome pool of all our samples. Extracted 16S rRNA and 18S rRNA sequences were matched against the SILVA database release 111 (Pruesse et al., 2007) using NCBI BLAST in blastn-mode v 2.2.29 (Altschul et al., 1997). The BLAST results were analyzed with the LCA algorithm of MEGAN v 5.10.6. (Huson et al., 2011) by assigning each SSU rRNA onto the predicted nodes of the NCBI taxonomy tree. In addition, a phylogenetic analysis was performed using the Maximum likelihood (ML)-based tree reconstruction algorithm of MLTreeMap v 2.061 (Stark et al., 2010) with default parameters. 16S/18S rRNA sequences were thereby added to the reference phylogeny of the GEBA tree of life (Wu et al., 2009).

For the whole metagenome similarity approach, all sequence reads were queried against the NCBI GenBank nt and nr databases by using NCBI BLAST in blastn- and blastx-modes, respectively. BLAST outputs were filtered by an *e*-value cutoff of 10^-5^ and a minimum-sequence-identity threshold of 60%. The diversity was then assessed based on the taxonomic affiliation of the matching sequences from the respective databases. Total read counts were computed for each hierarchy level in the NCBI taxonomy database using Fantom (Sanli et al., 2013). In the cases where the taxonomic rankings (e.g., genus or phylum) could not be detected for the matched species level taxonomy identifiers, the counts were added to the groups closest to the missing rankings. Relative abundances of taxonomic groups were calculated by normalizing read counts matching to the individual taxonomic group by the total number of read counts that satisfied the BLAST thresholds within each sample. The Chao1 index was calculated from the abundances of taxonomic annotations at the species level of the NCBI GenBank nr BLAST outputs, using the Vegan package (Oksanen et al., 2015) in R.

#### Functional Assignment and Analysis

Gene prediction was performed on the quality-trimmed sequence reads in FragGeneScan v 1.16 (Rho et al., 2010). Predicted gene sequences were subjected to blastp searches against the Swiss-Prot (Apweiler et al., 2004), KEGG Orthology (KO; Kanehisa and Goto, 2000), and CAZy (Lombard et al., 2014) databases. Protein domain searches were performed on the predicted gene sequences against the PFAM (Finn et al., 2010) and TIGRFAMs (Haft et al., 2003) databases by using HMMER v. 3.1b2 (Eddy, 1998) in hmmsearch mode. BLAST and HMMER outputs were filtered with an *e*-value cutoff of 10^-5^ and the BLAST outputs were further filtered by setting a minimum-sequence-identity threshold of 60%. Total read counts were calculated for each identifier in the respective database, and relative abundances were calculated by normalizing read counts matching to each database identifier in the corresponding functional database by the total number of read counts that satisfied the BLAST thresholds within each sample. Gene Ontology terms (Ashburner et al., 2000) were assigned based on the Swiss-Prot matches. Further annotation was done by utilizing the hierarchical structure of the KEGG Brite database where each protein match in the KO database was assigned to three more levels of biological organization according to the KB hierarchies. Abundance counts for each sample were averaged and mean relative abundances of the KO annotations were plotted for the four different hierarchy levels of the KB database by using CIRCOS v 0.63.4 (Krzywinski et al., 2009).

The KEGG analysis was elaborated by compiling literature on functional features related to the biofilm development cycle. Those functional features were then queried in the KB database, and the extracted KEGG functions were highlighted in the CIRCOS plot. Biofilm-related functions were derived according to the publications listed in Supplementary Table S2. The search terms used in the database queries included functions known to participate in the entire biofilm development cycle, such as initial biofilm attachment and adhesion; the full list of extracted KEGG functions can be found in Supplementary Table S2. Additionally, KEGG Mapper v 1.6 (Kanehisa et al., 2012) was used to map the orthologous enzyme- and protein-coding genes found in the periphyton metagenome onto the corresponding biochemical pathways under the broad KEGG category energy metabolism. Regarding energy metabolism, key enzymes of the carbon fixation pathways in prokaryotes were derived by extracting individual enzymes that are found only in the corresponding pathway module but not in other modules according to the functional hierarchy described by the KEGG Modules database.

Phylogenetic analysis results of the 16S rRNA gene extractions (Supplementary Figure S1) were used as references to select representative genomes from different phyla within the kingdoms of bacteria and archaea. Species with significant likelihood of metagenomic read placement in the MLTreeMap tree were selected for genome mapping. A representative genome of the same genus as the detected species was chosen in cases where the complete genome sequences of the detected species were not publicly available. Proteobacterial genomes were selected from the classes of *Alpha*-, *Beta*-, *Gamma*-, and *Delta*-*proteobacteria*. During the selection of the archaeal genomes, results of both the phylogenetic analysis and functional analysis of the energy metabolism (e.g., large number of reads mapping to methane metabolism) were taken into consideration. Eukaryotic genomes were disregarded from this analysis due to their substantially larger genome sizes. The complete list of species strains selected for the genome mapping for each phyla was as follows: *Bdellovibrio bacteriovorus* strain 109J, *Halorhodospira halophila* SL1, *Janthinobacterium* sp. Marseille, *Paracoccus denitrificans* PD1222, *Roseobacter denitrificans* OCh 114 (*Proteobacteria*), *Dehalococcoides* sp. BAV1, *Gloeobacter violaceus* PCC 7421, *Synechocystis* sp. PCC 6803, *Nostoc punctiforme* PCC 73102, *Anabaena variabilis* ATCC 29413 (*Cyanbobacteria*), *Parabacteroides distasonis* ATCC 8503, *Cytophaga hutchinsonii* ATCC 33406, *Bacteroides fragilis* NCTC 9343, *Flavobacterium columnare* ATCC 49512, *Gramella forsetii* KT0803 (*Bacteroidetes*), *Metallosphaera sedula* DSM 5348, *Methanocaldococcus jannaschii* DSM 2661, *Methanopyrus kandleri* AV19, *Halobacterium salinarum* R1, *Methanosarcina barkeri* str. Fusaro (archaea) (Supplementary Table S3). Metagenomic sequences were concatenated in a single FASTA file and were aligned to the corresponding genome by BLAST in blastn mode with an *e*-value threshold of 10^-5^. BRIG v. 0.95 (Alikhan et al., 2011) was used to visualize the metagenomic sequence alignments to the genomes with 100, 90 and 80% sequence identities. Genome mapping results were plotted in increasing order of total number of bases covered in the corresponding genome, from innermost to the outermost circular genomic maps drawn by BRIG. In addition to the genome mapping, pathways under the KB category energy metabolism were highlighted in the outermost circular genome map. Genomic loci of proteins and annotated COG functions were downloaded for each genome from the NCBI Genome database. COG functions were matched to their KEGG equivalents by id mapping and the loci belonging to the listed pathways were visualized by using BRIG.

## Results and Discussion

### Comparison of Samples

Overall, the taxonomic and functional composition of the six samples were found to be similar according to both the taxonomy assignments at the phylum-level and the second hierarchy level KB annotations (Supplementary Figures S2 and S3, respectively). Supplementary Figure S2 shows a slight difference in the microbial composition of the third site in comparison to the rest of the sites. This site is the deepest (**Table 1**) and is thus likely to have the lowest re-suspension of organisms from the bottom sediment, which might explain its somewhat deviant taxonomic composition. Since the overall taxonomic and functional content of the sampled communities were similar (Supplementary Figures S2 and S3), we performed the rest of the analyses on the averaged abundances of each taxonomic and functional group.

### Sequence Reads of Unknown Origin

We adopted several strategies for taxonomic and functional annotation, including the similarity searches against the databases of NCBI GenBank nt and nr, SILVA, Swiss-Prot, KO, CAZy, PFAM, and TIGRFAMs, but the percentage of reads of unknown origin – that is, sequences whose taxonomic and functional nature we were unable to deduce through sequence analysis – remained high (78.9%). As reviewed by Gilbert and Dupont (2011) and Bik (2014), high proportions of unknown reads are common in metagenomics studies of marine communities. However, most of these studies were performed on planktonic communities with a relatively small amount of eukaryotic organisms. Since the number of sequenced prokaryotic species is much higher than that of eukaryotic species, combined with the fact that eukaryotes have larger genomes with more non-coding DNA (Ahnert et al., 2008), it is reasonable to assume that a community composed of a substantial eukaryotic portion, such as periphyton, will contain a higher percentage of unknowns. This notion is also supported by the results of Allen et al. (2013) where planktonic communities from larger size fractions, which contain more eukaryotes, comprise a higher fraction of unknown sequences. An additional explanation may lie in sequences of viral origin in the metagenome. Results of the protein domain searches to the PFAM database show that reverse transcriptase (RNA-dependent DNA polymerase) domain (PF00078, PF07727) and integrase core domain (PF00078, PF07727), which are commonly found in viral proteins, are highly abundant in periphyton (Supplementary Table S4). Hence, periphytic sequences of unknown origin may originate from the large reservoir of undescribed viral genes (Wommack et al., 2015). The high percentage of unknown sequences in periphyton, either of eukaryotic, or viral origin, strongly indicates that periphyton organisms are not well represented in the public sequence repositories.

### Taxonomic Analysis

**FIGURE 1:**
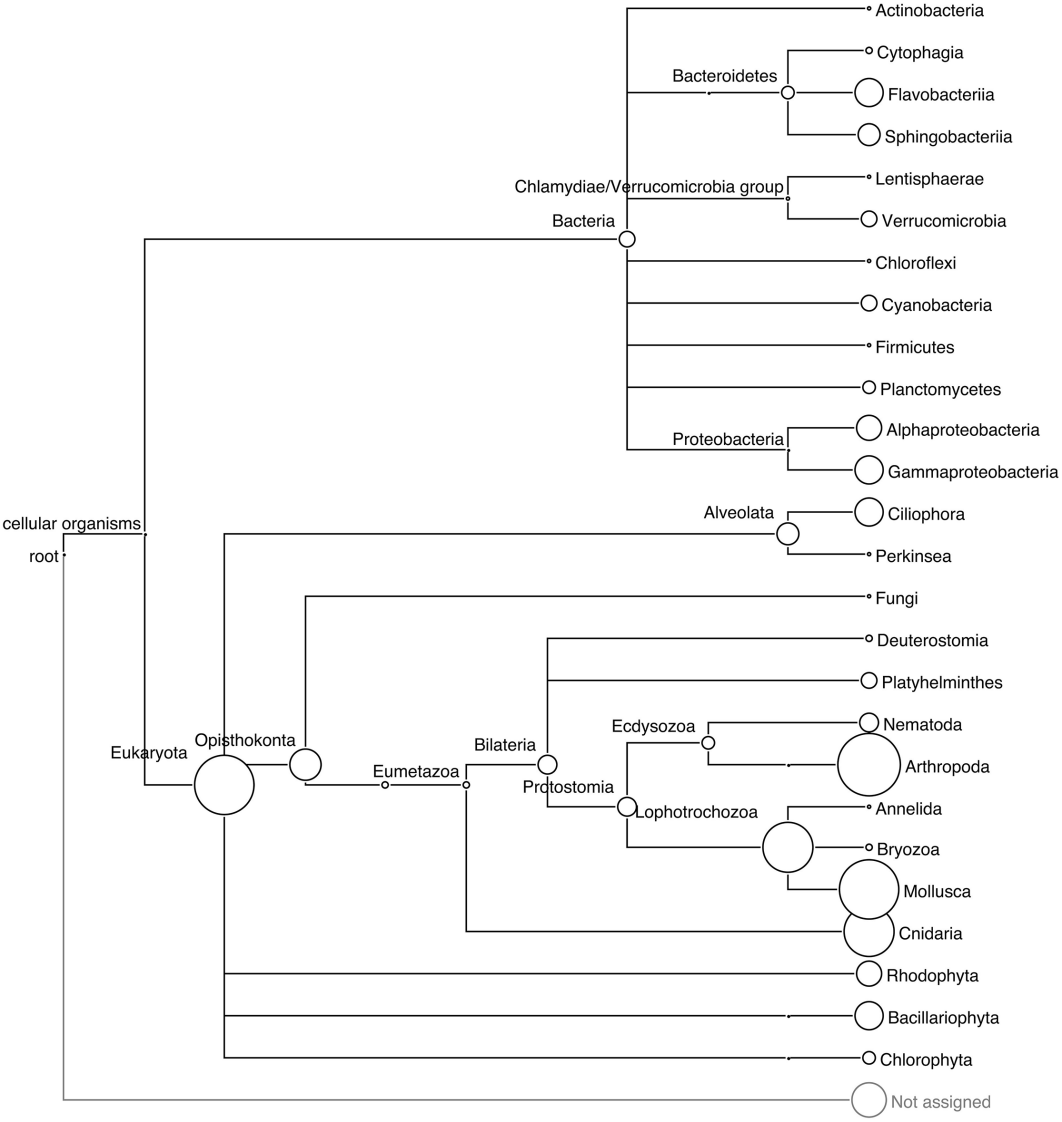
**Phylogenetic relationships among the taxa recovered in the periphyton samples.** LCA tree based on the BLAST matches of extracted 16S and 18S SSU rRNA gene sequences from periphyton metagenome in the SILVA database v 111. The size of the circles correspond to the abundance of the 16S and 18S sequences, and the circle size of the *Arthropoda* clade corresponds to 177 sequences.

The different approaches used to assess the taxonomic diversity of periphyton communities produced complementary views of the microbial composition. LCA and phylogeny-based SSU rRNA gene analysis supplemented each other and allowed us to obtain an overall view of marker gene-based diversity. From the 550,670 reads, a total of 751 eukaryotic and 67 bacterial SSU rRNA gene sequences were extracted and analyzed. In these metagenomes, with a high eukaryotic content and at the present sequencing depth, we found a low proportion of bacterial rRNA sequences. The low copy number of bacterial rRNA compared to that of eukaryotes (Klappenbach et al., 2001; Lee et al., 2009) is likely the cause of this low proportion of bacterial rRNA. Furthermore, the 18S copy numbers in eukaryotic genomes increase in a linear fashion with the genome sizes of the organisms throughout the eukaryotic part of the tree of life. Prokopowich et al. (2003) showed that the copy numbers of rRNA sequences varied between 39 and 26,048 in the genomes of 162 eukaryote species. **Figure 1** shows the taxonomic diversity of the extracted SSU sequences and their abundance according to the LCA algorithm of MEGAN. Eukaryotic invertebrate phyla such as *Arthropoda*, *Mollusca*, and *Cnidaria* made up the majority of all extracted SSU sequences. The LCA algorithm estimated the diversity of bacterial sequences by assigning reads to nine phyla, of which *Proteobacteria*, *Bacteroidetes*, *Verrucomicrobia*, and *Cyanobacteria* occurred in highest abundance. In addition, SSU sequences of phototrophic groups such as diatoms (*Bacillariophyta*) and red algae (*Rhodophyta*), and the protozoan group *Ciliophora*, were present in the community according to the LCA analysis.

The phylogenetic analysis results are shown in Supplementary Figure S1, which illustrates the placement of SSU sequences onto the built-in tree of MLTreeMap. The bacterial SSU sequences were mainly assigned to *Firmicutes*, *Cyanobacteria*, *Actinobacteria*, *Bacteroidetes*, and several subclades of *Proteobacteria*. One of the extracted SSU sequences was identified as related to *Metallosphaera sedula*, an archaeal extremophile species known for its extraordinary tolerance to heavy metals (Huber et al., 1989). Metagenomic sequence mapping of periphyton reads to the 2,191,517 base pairs long, complete genome sequence of this species resulted in a coverage of 0.03% (Supplementary Table S3) of the whole archaeal chromosome. Since *M. sedula* is known to live only in extreme environments including acid mines, volcanic fields and hot springs (Baker and Banfield, 2003) and the mapping of periphyton sequences was quite low, it is plausible that the identified 16S sequence belongs to a mesophilic crenarchaeotal relative of this species from the marine environment. MLTreeMap identified a large diversity of bacterial reads but did not assign as many sequences to the eukaryotic groups, *Arthropoda*, *Mollusca*, or *Cnidaria*, as expected from the LCA results. The eukaryotic placements should be interpreted bearing in mind the large fungal and the low metazoan representation in the eukaryotic part of the MLTreeMap tree.

Given the low sampling probability of the rRNA sequences in complex metagenomes and the variability in SSU copy numbers, the marker gene-based approaches to taxonomic identification should be viewed as a partial description both for the microbial diversity within the communities and for the abundance of organisms.

#### Community Structure from Whole Metagenome-based Similarity Searches

**FIGURE 2:**
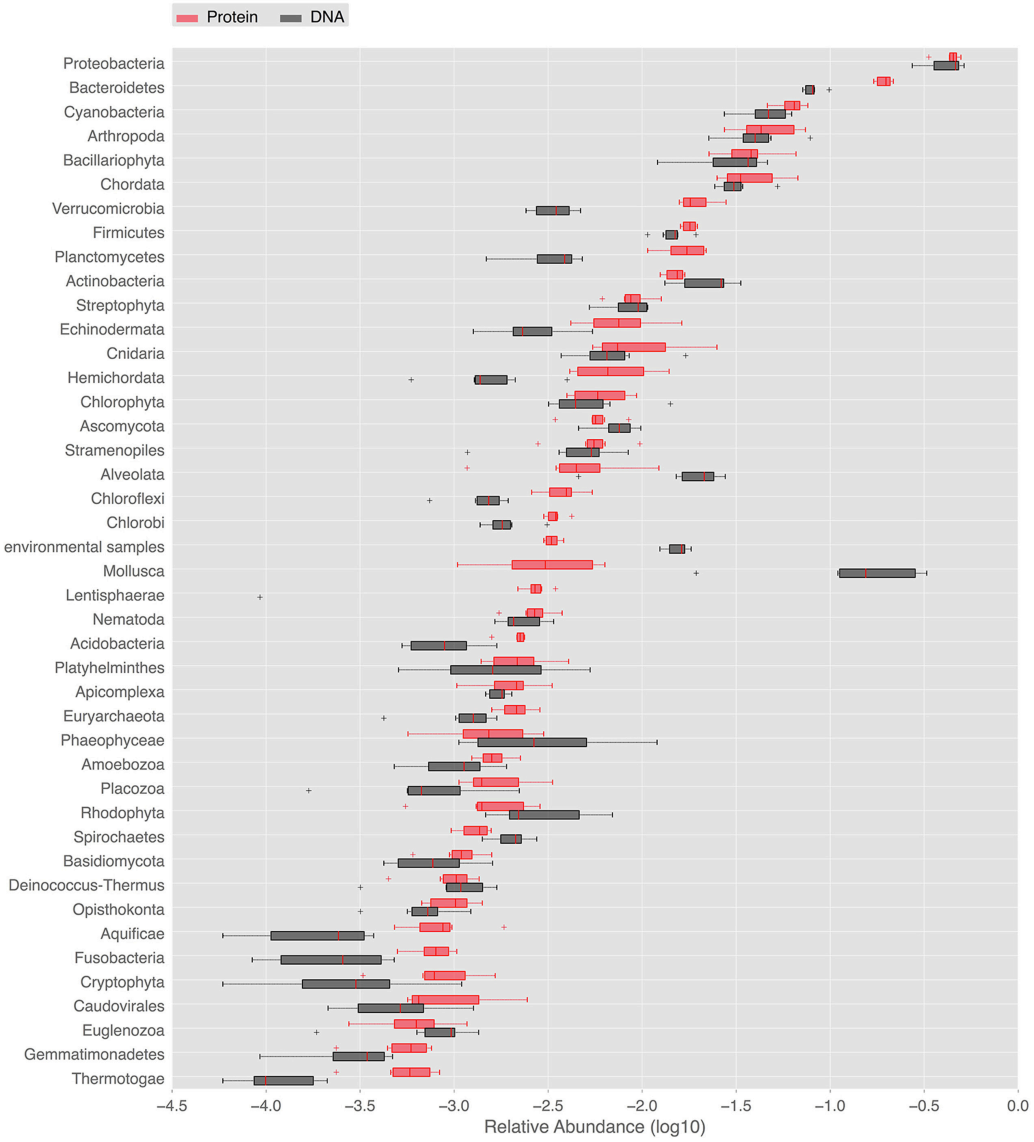
**Ranked abundances of phyla identified in the periphyton metagenome.** Relative abundances of phyla found in periphyton according to whole metagenome similarity searches to public databases. Red boxes labeled with “DNA” are the relative abundances of phyla according to the blastn matches in the NCBI nt database. Black boxes labeled with “Protein” are the relative abundances of phyla according to the blastx matches in the NCBI nr database.

In order to provide a complementary view on the taxonomic composition, whole metagenome-based similarity searches in the NCBI nt/nr databases were performed. They deepened the description of marine periphyton diversity and further illustrated the dominant groups. The NCBI nt and nr similarity searches gave 34,186, and 107,422 hits satisfying the BLAST thresholds, respectively. Using the NCBI nr annotations, as many as 33 eukaryal, 25 bacterial and 2 archeal phyla was detected in our periphyton samples. At the species level, the Chao1 index estimated 8375 species in all phyla. The abundant phyla found in our periphyton samples are shown in **Figure 2**. *Proteobacteria* was the most abundant phylum according to the BLAST hits against the nucleotide and protein databases. It was followed by *Bacteroidetes* and *Cyanobacteria* according to the protein matches. However, according to the nucleotide similarities the second most abundant taxa was the eukaryotic group *Mollusca*, followed by *Bacteroidetes*. Slightly different from the LCA results, there was a high number of protein matches to phototrophs including *Cyanobacteria*, *Bacillariophyta*, *Streptophyta*, and *Chlorophyta*. Additionally, around 25% of the proteobacterial matches were found to belong to *Roseobacter*, a phototrophic genus of *Alpha-proteobacteria*. Our results for periphyton bacterial abundance are roughly comparable to the results of planktonic bacteria sampled in the same region in the study of Dupont et al. (2014). These authors found that *Proteobacteria* dominated the Gullmar fjord surface waters (sample GS689), followed by *Bacteroidetes* and *Actinobacteria*. They also detected *Cyanobacteria*, *Verrucomicrobia*, and *Firmicutes*, and these findings are similar to the results presented in **Figure 2**. Since periphyton contains much more eukaryotic species than the planktonic communities studied by Dupont et al. (2014), there are also clear differences in community compositions between the periphyton described here and the planktonic communities described by Dupont et al. (2014).

Bacterial phyla identified by the SSU rRNA-based phylogenetic analysis were in line with results from the entire metagenome-based BLAST analysis against the NCBI databases. BLAST searches additionally showed high abundance of sequences belonging to eukaryotic phyla including *Chordata* and *Echinodermata* as well as the protozoan superphylum *Alveolata*. High numbers of significant matches to marine metazoans, within for example *Chordata*, *Arthropoda*, and *Mollusca*, is worth mentioning since biofilms are known to stimulate the settlement of larvae belonging to these eukaryotic groups (Unabia and Hadfield, 1999). The larvae of these organisms rely on the EPS matrix of the biofilm to secure the progression of their development cycle prior to metamorphosis. The biofilm provides firmer attachment than clean surfaces, such that the larvae are more protected against the shear forces of turbulent water (Zardus et al., 2008). This settlement process of eukaryotic larvae is pivotal for the development of hard fouling on ship hulls and marine installations, which is an issue of great economic and environmental importance (Abbott et al., 2000).

Many factors, such as habitat type, nutrient availability, and sampling season, as well as methodological factors such as sampling strategies and microbial detection methodology, complicate cross-study comparisons of periphyton communities. For example, the type of substrate for biofilm colonization has been debated. For freshwater periphyton, glass substrate has been questioned since communities developed on glass (or other artificial substrata) might misrepresent communities on natural substrata (Cattaneo and Amireault, 1992; Yang and Flower, 2012). However, Jackson et al. (2001) found the same richness in freshwater biofilms on glass as on natural substrata. For marine periphyton, comparisons of substrata are much more scarce, but Desrosiers et al. (2014) found no differences in diatom species richness between glass, plexiglas, and tiles. Also the detection methodology will strongly influence the description of the communities. Nevertheless, our protein sequence similarity-based abundance results are in agreement with fluorescent in situ hybridization-based findings from a river biofilm in a study that indicated the dominance of the bacterial phyla *Proteobacteria* and *Bacteroidetes* (Araya et al., 2003). Our findings also overlap with the 16S rRNA amplicon sequencing-based results of Kriwy and Uthicke (2011), where *Proteobacteria*, *Bacteroidetes*, and *Cyanobacteria* were identified as the prominent bacterial groups in periphyton of the Great Barrier Reef. In the present study *Bacillariophyta* and *Cyanobacteria* were identified as being among the most abundant phototrophic phyla in both marker gene analyses and whole metagenome-based similarity searches, which is supported by previous studies of marine periphyton (Shamsudin and Sleigh, 1995; Eriksson et al., 2009b). Finally, our results are in line with the findings of Azim et al. (2002) who showed that macro-invertebrate larvae are highly abundant in periphyton sampled from aquaculture ponds. Since metagenomics studies the entire gene pool of a community, theoretically circumventing the restrictions of the previous methodologies on diversity, we argue that this approach has the potential to cover the diversity of periphyton in an integrative manner subject to the use of sufficient sequencing depth.

### Periphyton Functions and Pathways

**FIGURE 3:**
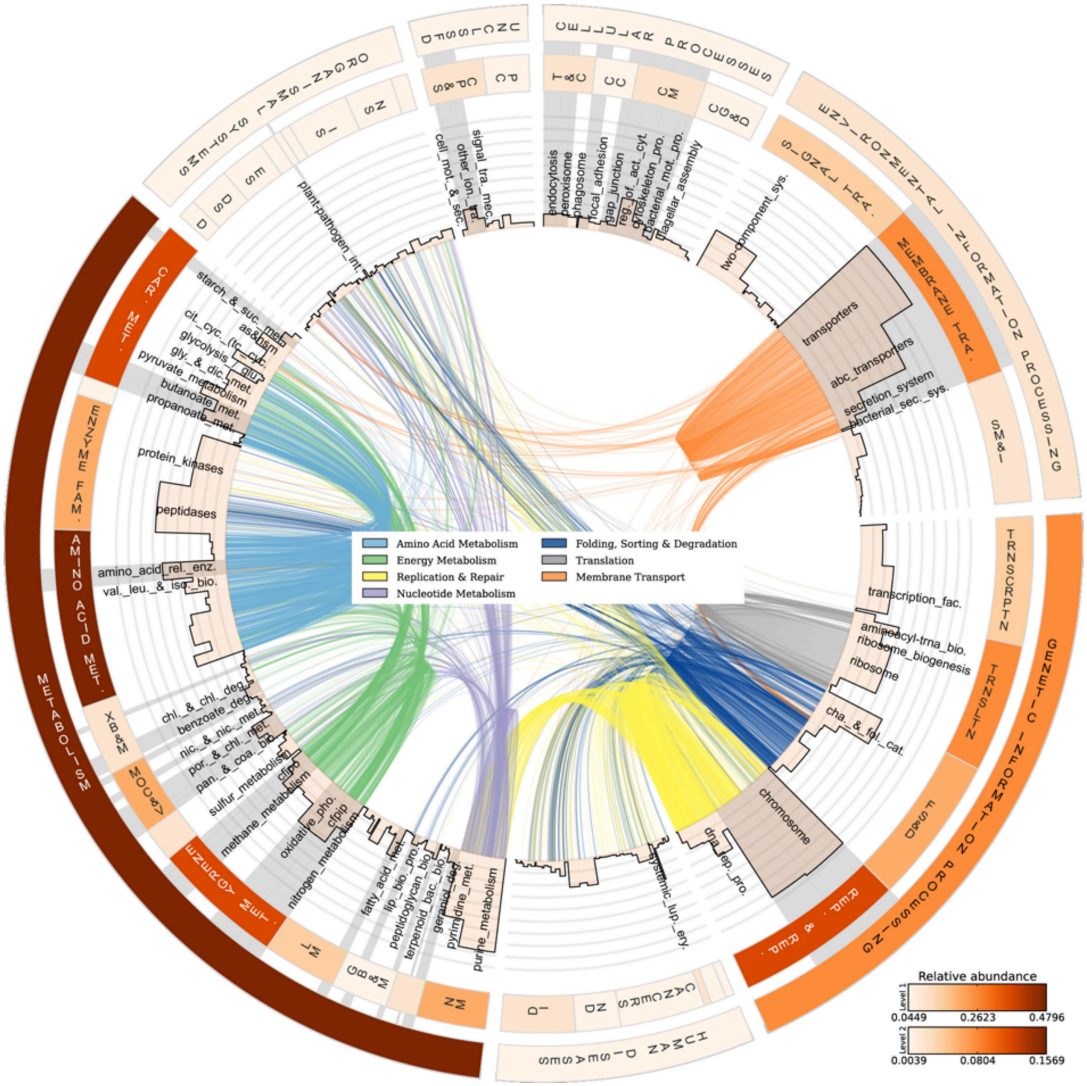
**Abundances and linkages of KEGG functions in periphyton.** CIRCOS plot based on the functional analysis of periphyton metagenome data by using the KB database as reference. The outer and inner circular heat maps shows the mean relative abundances for the broadest hierarchy level (Level 1) and the second hierarchy level in the KB database, respectively. The top and bottom color codes represent the relative abundances of the outer and inner circular heat maps, respectively. The bars in the innermost circle show the mean relative abundances of genes encoding proteins at the pathway level. The curved lines in the center represent the enzyme-pathway linkage information as indexed in KEGG. Genes encoding enzymes and proteins that are shared among different pathways are illustrated by these curved lines. Gray highlights on top of pathway names are the manually curated biofilm-related subcategories as defined in **Table 2**. Full names of the abbreviated KB categories shown in this plot can be found in Supplementary Table S13.

The analyzed communities contained a comprehensive set of functional genes and biochemical pathways. **Figure 3** summarizes the analysis of genes encoding functional proteins based on the hierarchical structure of the KB database. As shown by the heat map of the outermost circle in **Figure 3**, the abundances of genes encoding metabolism and genetic information processing were the highest among the broadest (level 1) KB categories. These two categories essentially comprise ubiquitously present functions in living cells and include carbohydrate metabolism, DNA replication and repair, and chromosomal proteins. Metabolic pathways native to carbohydrate metabolism, amino acid metabolism, energy metabolism, and nucleotide metabolism were highly abundant in the metagenome. In the broad functional group of genetic information processing, proteins involved in replication and repair and translation were very common. In the following, we have focused the discussion on the genes and functional groups that are of special interest for organisms living in a biofilm and those participating in energy metabolism and nutrient cycling.

#### Biofilm Indicators in Periphyton

Biochemical functions of interest for the biofilm mode of life are specified and described in Supplementary Table S2. The broadest KB categories, viz. cellular processes and environmental information processing, are of particular interest for the scrutiny of biofilm structure and function since these categories contain functions associated with surface adhesion and initial attachment as well as microbial interactions and EPS production (Supplementary Table S2 and references therein). The biofilm-relevant protein groups under cellular processes include those belonging to cell motility, cell communication and transport and catabolism (Supplementary Table S2), which had moderate numbers of matches to the periphyton metagenome. Although it is hard to assess the requirement of motility for periphytic organisms after the establishment of the biofilms, the fairly low abundance of genes encoding motility proteins in the metagenome (Supplementary Table S5) may be a result of the successive colonization by non-motile organisms throughout the biofilm development cycle. Transport and catabolism-related functions were also detected including the genes encoding proteins that form the phagosome and enzymes that take role in endocytosis, which could be important for protists that feed on bacteria in a biofilm.

Supplementary Table S6 lists the second hierarchy level categories in the KB database and their abundances in the periphyton metagenome. Apart from the prominent functions that are expected to be abundant such as amino acid metabolism, energy metabolism, or replication and repair, there are several more functional groups that are important for the organisms in a biofilm, including the moderately abundant xenobiotics biodegradation and metabolism. Periphyton biofilms are known to absorb heavy metals and metabolize pollutants including xenobiotics in the environment (Hahn and Hofle, 1999; Azim et al., 2005). The low abundance of glycan biosynthesis and metabolism is noticeable despite the anticipated structural role of glycans in the EPS matrix (Frolund et al., 1996). On the other hand, carbohydrate metabolism is the second most abundant category at this level of KB functions (Supplementary Table S6), which comprises enzymes potentially involved in the biosynthesis and degradation of EPS polysaccharides.

Among the functions belonging to the broad KB category of environmental information processing, membrane transport is both relevant to the biofilm mode of life and as shown in the inner heat map of **Figure 3**, found to be abundant in the metagenome. Membrane transport covers secretion systems, bacterial secretion systems, transporters, and ABC transporters, where the latter two are among the most abundant functions in the metagenome dataset (**Table 2**). Similar to these results, Ganesh et al. (2014) found that genes encoding adhesion, bacterial secretion, and antibiotic resistance were enriched in particle-associated compared to free-living marine microbial communities. The categories of ABC transporters and transporters share a substantial number of proteins in the KEGG database. In fact, the 205 different ABC transporters detected are all found in the category of transporters. The hierarchical classification of proteins in databases such as KB can be misleading when calculating abundances of individual pathway categories. One protein can belong to several broader-level categories such that it can be counted several times, which results in overestimation of the abundances of certain pathways in high-throughput data analyses. Although there are several methods proposed to account for the validity of the pathway abundances in metagenomic data (Ye and Doak, 2009; Sharon et al., 2011) and different studies have utilized a variety of *ad hoc* solutions, there is no established methodology to handle this problem. In our analyses we choose to illustrate this phenomenon as links among the categories in the center of **Figure 3**. These links show the association of an enzyme in different pathways, as indexed in KB, and visualize the level of overlap among the functional groups. For example, the membrane transport categories ABC transporters and transporters are highly connected. Since this topic is not within the scope of the current paper, we solely point to the problem here and argue that pathway abundance results within the hierarchical database context should be interpreted cautiously. Regarding the membrane transport category, 278 transport proteins were detected in total. Multiple drug resistance-related ABC transporters as well as various sugar, peptide, and lipid transport proteins were found to be the most abundant groups of transporters in the periphyton metagenome (Supplementary Table S7). Transporter protein domains are also predominant among the most abundant ten protein domains matched in the TIGRFAMs database (Supplementary Table S8).

**Table 2:** Biofilm-relevant pathways and protein complexes found in periphyton.

Pathway	Relative abundance	Pathway	Relative abundance
**Energy metabolism**	**0.15**	**Cell motility**	**0.037**
Oxidative phosphorylation	0.037	Cytoskeleton proteins	0.019
Carbon fixation pathways in prokaryotes	0.027	Bacterial motility proteins	0.0085
Methane metabolism	0.018	Regulation of actin cytoskeleton	0.0042
Nitrogen metabolism	0.017	Flagellar assembly	0.003
Photosynthesis proteins	0.015	Bacterial chemotaxis	0.0023
Photosynthesis	0.014	**Cell Communication**	**0.018**
Carbon fixation in photosynthetic organisms	0.013	Focal adhesion	0.0054
Sulfur metabolism	0.0069	Gap junction	0.0051
Photosynthesis – antenna proteins	0.00091	Tight junction	0.0038
**Membrane transport**	0.15	Adherens junction	0.0034
Transporters	0.072	**Transport and catabolism**	**0.024**
ABC transporters	0.05	Peroxisome	0.0065
Secretion system	0.016	Endocytosis	0.0061
Bacterial secretion system	0.0094	Phagosome	0.0058
Phosphotransferase system (PTS)	0.00074	Lysosome	0.0052

*Relative abundances of pathways and protein complexes under broader KB categories (in bold).*

#### Energy Metabolism and Elemental Cycling

Although metagenomics reflects potential rather than realized functional capacity, our data offered a window of discussion on the poorly known energy metabolism aspects of periphyton. According to the KEGG results, oxidative phosphorylation, and carbon fixation pathways in prokaryotes are the most abundant pathways under energy metabolism (**Table 2**). These findings support the taxonomic inferences from the whole metagenome-based similarity searches on hetero- and phototrophic inhabitants of periphyton, where the former group is mainly composed of *Bacteria*, *Arthropoda* and *Chordata* and the latter of *Cyanobacteria* (**Figure 2**). The relative abundances of pathway modules belonging to carbon fixation pathways in prokaryotes and the absolute read counts of genes encoding the key enzymes in those modules can be found in Supplementary Table S9. Among the bacterial primary producers, two carbon fixation strategies, namely the Calvin cycle and the rTCA, were found to be prominent. The Calvin cycle is known to be present in *Cyanobacteria*, algae and certain proteobacterial groups whereas the rTCA is known to exist in anoxygenic photosynthetic bacteria. Among the detected matches to the enzyme RuBisCO, 42 out of 44 matches were found to be of bacterial origin, mainly belonging to the classes of *Alpha-* and *Beta-proteobacteria*. Five reads matching the RuBisCO sequence were identified to be of cyanobacterial origin. Purple sulfur bacteria belonging to *Proteobacteria* and green sulfur bacteria of the phylum *Chlorobi* were the identified groups in our data that assimilate carbon through rTCA. The majority of the hits to the detected key enzymes of rTCA cycle, namely pyruvate-ferredoxin/flavodoxin oxidoreductase and pyruvate carboxylase, were found to be originated from *Proteobacteria* and *Firmicutes*. Genes encoding enzymes involved in other carbon fixation pathways including the 3-HP bicycle, the reductive acetyl-CoA pathway, the 3-HP/4-HB cycle and the DC/HB cycle were also found to have matches in the periphyton metagenome. However, genes encoding the key enzymes in these pathways could not be detected except for the identified single copy of the enzyme 2-methylfumaryl-CoA isomerase belonging to the 3-HP cycle (Supplementary Table S9). The methanogenic class of the phylum *Euryarchaeota*, *Methanomicrobia* can utilize the acetyl-CoA pathway for carbon fixation (Matschiavelli et al., 2012) and was also found in low abundances in the sampled periphyton. Hence, it is conceivable that the Calvin cycle and rTCA are not the only carbon assimilation pathways in the marine biofilms.

The nitrogen metabolism in periphyton is important since it has implications for the fate of the increased nitrogen load (eutrophication) in coastal ecosystems (Giblin et al., 2013). We identified various genes for enzymes catalyzing the steps of assimilatory and dissimilatory nitrate reduction as well as denitrification and nitrogen fixation (Supplementary Figure S4). Genes encoding the enzymes catalyzing assimilatory and dissimilatory reduction of nitrate to ammonia were all found to be present in the periphyton metagenome. The genes encoding the enzymes for the assimilatory nitrate reduction pathway mapped to a wide range of bacterial taxa, mainly belonging to alpha-proteobacterial groups including *Rhizobiales* and *Rhodobacterales*, and gamma-proteobacterial groups including *Enterobacteriales*, *Alteromonadales*, and *Pseudomonadales*. Dissimilatory nitrate reductase genes were mainly of alpha-proteobacterial origin, belonging to the order *Rhodobacterales*. Furthermore, we found that *denitrification* may be an important process in the periphyton communities, since the majority of the genes encoding enzymes carrying out the individual steps of denitrification could be detected in the metagenome dataset. The denitrification reactions are carried out almost exclusively by proteobacterial orthologs of the corresponding enzymes. An interesting exception in terms of functional gene diversity throughout the denitrification pathway is the *nos*Z gene, which has high similarity only to *Flavobacterial* orthologs (Supplementary Table S10). This gene encodes the enzyme nitrous-oxide (N_2_O) reductase that catalyzes the final reaction step of denitrification under anaerobic conditions. Truncation of denitrification at this step results in the emission of N_2_O, a greenhouse gas, to the atmosphere (Zumft, 1997). Although the diversity of organisms with the N_2_O reduction capability is restricted to the order *Flavobacteriales* within periphyton, this group is the second most abundant order in the studied biofilm communities and constitutes 11% of the total protein matches. Hence, the N_2_O reduction capability of periphyton is secured by this highly abundant bacterial population. Moreover, detection of the denitrification process in periphyton, which requires anaerobic conditions, is supported by Simon et al. (2014) who reviewed metagenomics studies of marine particle-attached communities and found evidence of low-oxygen micro-zones in attached communities. Such microzones has also been detected and coupled to high denitrification potential in freshwater periphyton (Ishida et al., 2008). We also detected genes encoding enzymes responsible for nitrogen fixation belonging to the members of the orders *Rhizobiales* and *Methylococcales*. However, we did not detect any genes for ammonia oxidizing enzymes that initiate either the aerobic nitrification reactions or the anaerobic annamox reactions.

According to both KEGG (**Table 2**) and Gene Ontology analysis results (Supplementary Table S11), metabolism of one-carbon compounds was among the most abundant biochemical processes. In the KEGG analysis, these processes are represented by a large number of sequences mapping to methane metabolism by which both the formation and utilization of those one-carbon compounds are mediated (Supplementary Table S12). In order to further study the utilization of complex sugar polymers, BLAST hits to the five carbohydrate active enzyme classes found in the CAZy database were analyzed and are listed in **Table 3**. Relatively higher counts of Glycoside Hydrolase (GH) and Glycosyl Transferase (GT) families point toward a large capacity of enzymes carrying out the degradation and biosynthesis of complex sugars (Edwards et al., 2010). **Table 3** also lists the individual CAZy families where the GH Family 13 is found to be the most abundant. Enzymes belonging to this family target various polysaccharides including starch and glycogen. The EPS matrix is known to comprise such long carbohydrate molecules, and it constitutes a carbon source for the biofilm communities. Both GT family 35 and CBM family 48 participate in glycogen metabolism. They were found among the most abundant CAZy families, supporting the ubiquitous degradation of glycogen-like polysaccharides found in the EPS matrix. There is also a substantial potential for degradation of chitin-like polymers in the periphyton metagenome, as inferred from the high numbers of Carbohydrate Esterase (CE) family 4 and Carbohydrate Binding Module (CBM) family 50 gene copies. This is likely due to the presence of arthropods and mollusks, who excrete chitin in the biofilms. The hydrolysis of complex-polysaccharide structures by the listed CAZy families followed by the decomposition of fatty acid molecules, yield the acetate source required for the methanogens in the community. Although methanogens can utilize CO_2_ and methylamine/dimethylamine/trimethylamine compounds as electron acceptors, genes encoding enzyme orthologs belonging to the acetate module of the methanogenesis pathway are the most abundant in the periphyton metagenome. Many orthologs involved in methane metabolism was found, but the *Methanomicrobia* class of the phylum *Euryarchaeota* was the only previously described methanogenic group found in the biofilm communities (Supplementary Table S12).

**Table 3:** CAZy analysis results.

Class	Total # of hits	Family	Total # of hits
GT	8625	GT35	2462
		GT51	1570
		GT2	1479
		GT4	1055
GH	7716	GH13	2560
		GHnc	841
CE	2674	CE4	1170
		CE11	717
CBM	1528	CBM50	645
		CBM48	600
PL	185		

*Total number of BLAST hits from the periphyton metagenome to the five broad CAZy classes Glycosyl Transferase (GT), Glycoside Hydrolase (GH), Carbohydrate Esterase (CE), Carbohydrate Binding Module (CBM) and Polysaccharide Lyase (PL) and to the 10 most abundant CAZy families under the classes GT, GH, CE, and CBM.*

As the pathways of energy metabolism described above may distinctively drive the energy supply of exclusive microbial groups, an exceptional versatility of metabolic pathways is operated by certain bacterial populations to yield energy. Representative genomes were selected to illustrate this phenomenon among the periphyton communities. For example, Supplementary Figure S5A shows that among the selected representative genomes, *Roseobacter denitrificans*, unlike others, utilizes a variety of pathways for its energy metabolism. Under oxic conditions and in the presence of light, this organism performs aerobic anoxygenic phototrophy (Tang et al., 2009). It can also use nitrate and trimethylamine N-oxide as electron acceptors in the absence of oxygen and light, thereby switching the route of its energy yielding pathways toward denitrification and methylotrophy. Periphyton metagenomic reads also cover a large portion of the *R. denitrificans* genome (Supplementary Table S3). The high abundance of genes encoding enzymes belonging to the methanotrophic pathways in the metagenome could partially be explained by the high abundance of this organism. Not surprisingly, the genus *Roseobacter* has been found to reach abundances up to 25% of the microbial community in the coastal marine environment (Wagner-Dobler and Biebl, 2006). In contrast to the flexibility of *R. denitrificans* in energy metabolism, the genome of the detected cyanobacterial species *Anabaena variabilis* is rich in photosynthetic operons (Supplementary Figure S5B; Supplementary Table S3). Moreover, the genome of the methanogenic archaea *Methanosarcina barkeri* (Supplementary Figure S5D), belonging to the identified class of *Methanomicrobia*, contains the largest proportion of loci involved in methane metabolism among the selected genomes (Supplementary Table S3). Although only 0.27% of this genome was covered by the periphyton metagenomic reads, (1) the identified archaeal 16S rRNA sequence, (2) protein matches to the *Euryarchaeota*, (3) the detected methanomicrobial orthologs of the enzymes taking role in methane metabolism (Supplementary Table S12), and (4) the indication of low oxygen micro-zones, supported by the detection of denitrification related genes, all hint towards the existence of archaea in the studied periphyton communities. All in all, the identified pathways in energy metabolism may individually belong to specific groups of organisms such as the denitrifiers or the methanogens whereas there are also certain bacterial groups such as the *Roseobacter* clade that can utilize a multitude of the identified energetic pathways.

## Conclusion

We conclude that marine periphyton communities harbor a vast diversity of organisms and metabolic strategies. The high percentage of sequences of unknown origin indicates that metagenomic sequencing of communities with large proportions of eukaryotes requires even higher sequencing depth (or attempts at separation of eukaryotes or prokaryotes, if only one of the groups is targeted). It also indicates that periphyton organisms are under-represented in sequence repositories. Even so, the studied metagenomes signal the attached life-style of these communities, including genes encoding proteins involved in surface adhesion and EPS production. The detection of both aerobic and anaerobic pathways indicates the presence of physiochemical micro-zones where different metabolic processes take place. We did not only find a community that contains a great variety of strategies for energy metabolism but we also found specific species that encompass a multitude of energetic pathways. Although the sequencing effort in this study provides a limited coverage of all genomes in these marine biofilm communities, the metagenomics approach supplied a more integrative and detailed analysis of marine periphyton compared to previous descriptions.

## Conflict of Interest Statement

The authors declare that the research was conducted in the absence of any commercial or financial relationships that could be construed as a potential conflict of interest.

## Abbreviations

CBM: Carbohydrate Binding Module
CE: Carbohydrate Esterase
DC/HB: Dicarboxylate-hydroxybutryate
EPS: Extracellular Polymeric Substances
GH: Glycoside Hydrolase
GT: Glycosyl Transferase
3-HP: 3-Hydroxypropionate
3-HP/4-HB: 3Hydroxypropionate/4-Hydroxybutyrate
KB: KEGG Brite
LCA: Lowest Common Ancestor
ML: Maximum Likelihood
PL: Polysaccharide Lyase
rTCA: Reductive Tricarboxylic Acid Cycle
SSU: Small Subunit
