# Supplementary material for: Metagenomic sequencing of marine periphyton: taxonomic and functional insights into biofilm communities
Source: Front Microbiol. 2015 Oct 30;6:1192. doi: 10.3389/fmicb.2015.01192 (PMC4626570; doi:10.3389/fmicb.2015.01192)
Supplement: Supplementary file 1 [file Data_Sheet_1.DOCX]

***Supplementary Material***



**Metagenomic Sequencing of Marine Periphyton: Taxonomic and Functional Insights into Biofilm Communities**

**Kemal Sanli^1^, Johan Bengtsson-Palme^2^, R. Henrik Nilsson^1^, Erik Kristiansson^3^, Magnus Alm Rosenblad^4^, Hans Blanck^1^ and K. Martin Eriksson^5*^**

^1^ Department of Biological and Environmental Sciences, University of Gothenburg, Gothenburg, Sweden

^2^ Department of Infectious Diseases, Institute of Biomedicine, Sahlgrenska Academy, University of Gothenburg, Gothenburg, Sweden

^3^ Department of Mathematical Sciences, Chalmers University of Technology, Gothenburg, Sweden

^4^ Department of Chemistry and Molecular Biology, University of Gothenburg, Gothenburg, Sweden

^5^ Department of Shipping and Marine Technology, Chalmers University of Technology, Gothenburg, Sweden

*** Correspondence**

Dr. K. Martin Eriksson

Department of Shipping and Marine Technology

Chalmers University of Technology

Hörselgången 4,

Gothenburg, 41296, Sweden

martin.eriksson@chalmers.se

1. **Supplementary Figures and Tables**

## Supplementary Tables

**Supplementary Table S1.** Physico-chemical data for sea water from a site (latitude 58.25830, longitude 11.43330) in the sampled region. Metadata for two depths (0 and 5 m, i.e. just above and just below the periphyton sampling depth of 1.5 m) during the time of periphyton colonization are given. The periphyton communities were sampled the 28^th^ of April, 23^rd^ of July, 30^th^ of August and 21^st^ of September 2004. Since the periphyton communities colonized the sampled substrate for 2.5 weeks before sampling, the metadata sampling represent the conditions during periphyton colonization. The physico-chemical data was downloaded from the Swedish Meteorological and Hydrological Institute (<http://www.smhi.se/klimatdata/oceanografi/havsmiljodata/marina-miljoovervakningsdata>) 2015-09-11.

| **Metadata sampling date** | **Metadata sampling depth (m)** | **Secchi depth (m)** | **Temperature (C°)** | **Salinity (PSU)** | **Dissolved oxygen concentration (ml/l)** | **Phosphate concentration (µmol/l)** | **Total phosphorous concentration (µmol/l)** | **Nitrite concentration (µmol/l)** | **Nitrate concentration (µmol/l)** | **Ammonium concentration (µmol/l)** | **Total nitrogen concentration (µmol/l)** | **Silicate concentration (µmol/l)** |
| --- | --- | --- | --- | --- | --- | --- | --- | --- | --- | --- | --- | --- |
| 19^th^ April | 0 | 6 | 7.7 | 21.6 | 7.83 | 0.07 | 0.41 | 0.11 | 3.19 | 0.24 | 25.4 | 8.3 |
| 19^th^ April | 5 | 6 | 7.1 | 22.9 | 7.83 | 0.05 | 0.36 | 0.06 | 0.87 | 0.14 | 18.8 | 3.2 |
| 7^th^ July | 0 | 9 | 16.0 | 26.1 | 6 | 0.04 | 0.34 | 0.03 | 0.1 | 0.14 | 12.8 | 1.1 |
| 7^th^ July | 5 | 9 | 16.0 | 26.1 | 6 | 0.04 | 0.41 | 0.02 | 0.1 | 0.14 | 14.4 | 1.1 |
| 20^th^ August | 0 | 9 | 19.1 | 22.3 | 5.58 | 0.08 | 0.42 | 0.05 | 0.1 | 0.23 | 15.8 | 1.4 |
| 20^th^ August | 5 | 9 | 19.1 | 22.3 | 5.67 | 0.09 | 0.42 | 0.04 | 0.1 | 0.2 | 17.1 | 1.4 |
| 8^th^ September | 0 | 9 | 17.1 | 22.2 | 6.68 | 0.07 | 0.49 | 0.03 | 0.1 | 0.36 | 20.9 | 0.3 |
| 8^th^ September | 5 | 9 | 17.1 | 26.0 | 5.16 | 0.08 | 0.36 | 0.02 | 0.1 | 0.25 | 14.3 | 1.3 |
| 20^th^ September | 0 | 5 | 15.3 | 27.3 | 5.87 | 0.09 | 0.5 | 0.04 | 0.1 | 0.23 | 14.8 | 0.9 |
| 20^th^ September | 5 | 5 | 15.3 | 27.3 | 5.9 | 0.09 | 0.55 | 0.05 | 0.1 | 0.34 | 15.9 | 0.8 |

**Supplementary Table S2.** Biofilm related functions. Pathways and protein groups relevant for the biofilm mode of life in the KEGG Brite database.

| **KEGG Brite Level1** | **KEGG Brite Level 2** | **KEGG Pathways** | **Role in biofilm** | **Reference** |
| --- | --- | --- | --- | --- |
| Metabolism | Carbohydrate Metabolism | Propanoate metabolism, Butanoate metabolism, Starch and sucrose metabolism | EPS - Extracellular polysaccharide production | (Frolund et al., 1996) |
| Metabolism | Amino Acid Metabolism | Amino acid related enzymes | EPS - Extracellular protein production | (Krohn-Molt et al., 2013) |
| Metabolism | Replication and Repair | DNA repair and recombination proteins, Chromosome | Microbial interactions - Horizontal Gene Transfer | ([Sorensen et al., 2005](#_ENREF_12)) |
| Metabolism | Energy Metabolism | Carbon fixation pathways in prokaryotes, Methane metabolism, Carbon fixation in photosynthetic organisms, Nitrogen metabolism, Sulfur metabolism | Microbial interactions - Elemental cycling | (Canfield et al., 2005) |
| Metabolism | Glycan Biosynthesis and Metabolism | Peptidoglycan biosynthesis, Lipopolysaccharide biosynthesis proteins | EPS - Extracellular polysaccharide production | (Frolund et al., 1996) |
| Metabolism | Metabolism of Cofactors and Vitamins | One carbon pool by folate, Porphyrin and chlorophyll metabolism, Pantothenate and CoA biosynthesis, Ubiquinone and other terpenoid-quinone biosynthesis, Nicotinate and nicotinamide metabolism | Microbial interactions | (Krohn-Molt et al., 2013) |
| Metabolism | Lipid Metabolism | Fatty acid metabolism | EPS - Export of cell components | (Flemming and Wingender, 2010) |
| Metabolism | Xenobiotics Biodegradation and Metabolism | Benzoate degradation, Drug metabolism - other enzymes, Chloroalkane and chloroalkene degradation | EPS - Sorption of xenobiotics | (Flemming and Wingender, 2010) |
| Metabolism | Metabolism of Terpenoids and Polyketides | Terpenoid backbone biosynthesis, Tetracycline biosynthesis, Polyketide sugar unit biosynthesis, Geraniol degradation | Microbial interactions | (Pang et al., 2012) |
| Cellular Processes | Transport and Catabolism | Phagosome, Peroxisome, Endocytosis | Microbial interactions | (Hahn and Hofle, 1999) |
| Cellular Processes | Cellular Processes and Signaling | Cell motility and secretion, Other ion-coupled transporters, Signal transduction mechanisms | Adhesion and initial attachment | - (Pasmore and Costerton, 2003) |
| Cellular Processes | Cell Communication | Gap junction, Focal adhesion | Adhesion and initial attachment | (Borlee et al., 2010) |
| Cellular Processes | Cell Motility | Cytoskeleton proteins, Bacterial motility proteins, Regulation of actin cytoskeleton, Flagellar assembly, Bacterial chemotaxis | Adhesion and initial attachment | (Barken et al., 2008) |
| Environmental Information Processing | Environmental Adaptation | Plant-pathogen interaction | Microbial interactions | (Givskov et al., 1996) |
| Environmental Information Processing | Membrane Transport | Transporters, ABC transporters, Secretion system, Bacterial secretion system | EPS production, resistance to biocides | (Gallaher et al., 2006) |

Supplementary Table S3. Metagenomic read mapping to bacterial and archaeal genomes. Metagenomic sequence read mapping to the complete genome sequences of the selected strains from the bacterial phyla *Protoebacteria*, *Bateroidetes*, *Cyanobacteria* and the archeal phyla *Euryarchaeota* and *Crenarchaeota.* The plot order refers to the plot order in Supplementary Figure S5, from innermost to outermost genome. Genome coverages by the periphyton metagenome reads were listed as total number of base pairs and as percent. Number of loci found in each genome annotated under the KEGG Brite category energy metabolism are also listed as belonging to the pathways: carbon fixation pathways in prokaryotes (CFPIP), photosynthesis (PS), oxidative phosphorylation (OP), methane (MM), nitrogen (NM) and sulfur metabolism (SM).

| **Phylum** | **Plot Order** | **Species strain name** | **Genome size (bp)** | **Genome coverage (total base pairs - %)** | **Number of loci annotated with energy metabolism** | | | | | |
| --- | --- | --- | --- | --- | --- | --- | --- | --- | --- | --- |
|  |  |  |  |  | **CFPIP** | **PS** | **OP** | **MM** | **NM** | **SM** |
| Proteobacteria | 1 | Bdellovibrio bacteriovorus strain 109J | 3,830,427 | 36,772 - 0.96 | 45 | 11 | 42 | 37 | 40 | 6 |
|  | 2 | Halorhodospira halophila SL1 | 2,678,452 | 42,855 - 1.60 | 41 | 12 | 46 | 57 | 50 | 11 |
|  | 3 | Janthinobacterium sp. Marseille | 4,110,251 | 59,599 - 1.45 | 12 | 14 | 59 | 70 | 65 | 53 |
|  | 4 | Paracoccus denitrificans PD1222 | 2,852,282 | 123,219 - 4.32 | 60 | 3 | 38 | 66 | 43 | 7 |
|  | 5 | Roseobacter denitrificans OCh 114 | 4,133,097 | 124,406 - 3.01 | 14 | 11 | 53 | 93 | 99 | 81 |
|  |  | Average | 3,520,902 | 77,370 - 2.27 | 34.4 | 10.2 | 47.6 | 64.6 | 59.4 | 31.6 |
| Cyanobacteria | 1 | Dehalococcoides sp. BAV1 | 1,341,892 | 14 224 - 1.06 | - | - | - | - | - | - |
|  | 2 | Gloeobacter violaceus PCC 7421 | 4,659,019 | 29 352 - 0.63 | 35 | 35 | 63 | 46 | 44 | 13 |
|  | 3 | Synechocystis sp. PCC 6803 | 3,569,561 | 49 260 - 1.38 | 10 | 29 | 68 | 50 | 45 | 31 |
|  | 4 | Nostoc punctiforme PCC 73102 | 8,234,322 | 75 756 - 0.92 | 52 | 46 | 77 | 86 | 77 | 17 |
|  | 5 | Anabaena variabilis ATCC 29413 | 6,365,727 | 87 210 - 1.37 | 42 | 38 | 98 | 76 | 78 | 18 |
|  |  | Average | 4,834,104 | 51 160 - 1.07 | 34.75 | 37 | 76.5 | 64.5 | 61 | 19.75 |
| Bacteroidetes | 1 | Parabacteroides distasonis ATCC 8503 | 4,811,379 | 48 595 - 1.01 | 39 | 8 | 22 | 59 | 41 | 12 |
|  | 2 | Cytophaga hutchinsonii ATCC 33406 | 4,433,218 | 67 385 - 1.52 | 35 | 8 | 50 | 35 | 41 | 11 |
|  | 3 | Bacteroides fragilis NCTC 9343 | 5,205,140 | 69 228 - 1.33 | - | - | - | - | - | - |
|  | 4 | Flavobacterium columnare ATCC 49512 | 3,162,432 | 107 839 - 3.41 | - | - | - | - | - | - |
|  | 5 | Gramella forsetii KT0803 | 3,798,465 | 112 814 - 2.97 | 45 | 9 | 33 | 48 | 53 | 14 |
|  |  | Average | 4,282,127 | 81 172 - 2.05 | 39.7 | 8.33 | 35 | 47.33 | 45 | 12.33 |
| Crenarchaeota | 1 | Metallosphaera sedula DSM 5348 | 2,191,517 | 657 - 0.03 | 80 | 9 | 51 | 96 | 43 | 6 |
| Euryarchaeota | 2 | Methanocaldococcus jannaschii DSM 2661 | 1,664,970 | 3 496 - 0.21 | 24 |  | 16 | 113 | 23 | 2 |
|  | 3 | Methanopyrus kandleri AV19 | 1,694,969 | 4 237 - 0.25 | 23 | - | 11 | 113 | 23 | - |
|  | 4 | Halobacterium salinarum R1 | 2,000,962 | 12 806 - 0.64 | - | - | 4 | - | 1 | - |
|  | 5 | Methanosarcina barkeri str. Fusaro | 4,837,408 | 13 061 - 0.27 | 43 | 20 | 44 | 171 | 80 | 7 |
|  |  | Average | 2,477,965 | 6 852 - 0.28 | 42.5 | 14.5 | 25.2 | 123.25 | 34 | 5 |

**Supplementary Table S4.** Abundances of PFAM domains. Relative abundances and absolute counts of the top 10 periphyton protein domains found in the PFAM database.

| **PFAM description** | **PFAM accession** | **Relative abundance** | **Absolute counts** |
| --- | --- | --- | --- |
| Tetratricopeptide repeat | PF07719, PF00515, PF13424, PF13181, PF13428, PF13432, PF13431, PF13176, PF13374, PF13174, PF13371, PF07721, PF13512 | 0.06 | 5067 |
| Reverse transcriptase (RNA-dependent DNA polymerase) | PF00078, PF07727 | 0.03 | 2485 |
| Ankyrin repeat | PF00023, PF13606 | 0.02 | 2096 |
| Ankyrin repeats (many copies) | PF13637, PF13857 | 0.02 | 1732 |
| TPR repeat | PF13414 | 0.01 | 768 |
| Integrase core domain | PF00665, PF13683, PF13333 | 0.01 | 749 |
| Methyltransferase domain | PF13847, PF08241, PF12847, PF13489, PF08242, PF13649, PF13659, PF13578, PF13679, PF13383, PF13708 | 0.01 | 746 |
| Leucine rich repeat | PF13855, PF13504 | 0.01 | 652 |
| Ankyrin repeats (3 copies) | PF12796 | 0.01 | 647 |
| Leucine Rich repeats (2 copies) | PF12799 | 0.01 | 561 |

**Supplementary Table S5.** Bacterial motility proteins. Relative abundance and absolute counts of proteins under the KEGG Brite category Bacterial motility proteins found in periphyton*.*

| **Protein** | **Relative abundance** | **Absolute counts** |
| --- | --- | --- |
| type IV pilus assembly protein PilB | 0.00058 | 22 |
| twitching motility protein PilT | 0.00051 | 17 |
| two-component system, NtrC family, response regulator PilR | 0.00046 | 16 |
| flagellar biosynthesis protein FlhA | 0.00044 | 17 |
| pilus assembly protein CpaF | 0.00038 | 16 |
| methyl-accepting chemotaxis protein | 0.00038 | 14 |
| flagellin | 0.00028 | 10 |
| two-component system, chemotaxis family, CheB/CheR fusion protein [EC:2.1.1.80 3.1.1.61] | 0.00027 | 11 |
| two-component system, chemotaxis family, sensor kinase CheA [EC:2.7.13.3] | 0.00023 | 9 |
| type IV pilus assembly protein PilM | 0.00021 | 9 |

Supplementary Table S6. KEGG Brite level 2 functions. Relative abundances and absolute counts of KEGG Brite hierarchy level 2 categories in periphyton.

| **Pathway** | **Relative abundance** | **Absolute counts** |
| --- | --- | --- |
| Amino Acid Metabolism | 0.24 | 8462 |
| Carbohydrate Metabolism | 0.21 | 7476 |
| Replication and Repair | 0.18 | 6148 |
| Energy Metabolism | 0.15 | 5343 |
| Membrane Transport | 0.15 | 5360 |
| Translation | 0.11 | 3839 |
| Folding, Sorting and Degradation | 0.087 | 3092 |
| Nucleotide Metabolism | 0.083 | 2981 |
| Lipid Metabolism | 0.072 | 2612 |
| Signal Transduction | 0.066 | 2309 |
| Xenobiotics Biodegradation and Metabolism | 0.061 | 2241 |
| Metabolism of Cofactors and Vitamins | 0.058 | 2084 |
| Enzyme Families | 0.057 | 1970 |
| Transcription | 0.049 | 1720 |
| Metabolism of Other Amino Acids | 0.043 | 1518 |
| Cellular Processes and Signaling | 0.038 | 1398 |
| Metabolism of Terpenoids and Polyketides | 0.038 | 1375 |
| Cell Motility | 0.037 | 1255 |
| Signaling Molecules and Interaction | 0.032 | 1060 |
| Glycan Biosynthesis and Metabolism | 0.031 | 1098 |
| Cell Growth and Death | 0.03 | 1008 |
| Transport and Catabolism | 0.024 | 825 |
| Cell Communication | 0.018 | 553 |
| Poorly Characterized | 0.017 | 603 |
| Biosynthesis of Other Secondary Metabolites | 0.015 | 529 |
| Environmental Adaptation | 0.0062 | 226 |
| Development | 0.005 | 152 |

Supplementary Table S7. Transporter proteins. Relative abundances and absolute counts of transporter proteins. Absolute counts and the relative abundances of the top 20 transporter proteins in periphyton.

| **Pathway** | **Relative abundance** | **Absolute counts** |
| --- | --- | --- |
| ATP-binding cassette, subfamily B, bacterial | 0.0028 | 102 |
| peptide/nickel transport system ATP-binding protein K02032 | 0.0027 | 100 |
| multiple sugar transport system ATP-binding protein | 0.0017 | 62 |
| peptide/nickel transport system permease protein K02034 | 0.0014 | 58 |
| multiple sugar transport system permease protein K02025 | 0.0014 | 50 |
| ATP-binding cassette, subfamily B, bacterial MsbA [EC:3.6.3.-] | 0.0014 | 48 |
| ABC-2 type transport system ATP-binding protein | 0.0013 | 46 |
| simple sugar transport system permease protein | 0.0013 | 45 |
| peptide/nickel transport system substrate-binding protein | 0.0013 | 47 |
| peptide/nickel transport system permease protein | 0.0012 | 46 |
| antibiotic transport system ATP-binding protein | 0.0011 | 39 |
| peptide/nickel transport system ATP-binding protein | 0.0011 | 40 |
| sulfonate/nitrate/taurine transport system ATP-binding protein | 0.001 | 38 |
| sn-glycerol 3-phosphate transport system ATP-binding protein [EC:3.6.3.20] | 0.001 | 36 |
| multiple sugar transport system substrate-binding protein | 0.00099 | 33 |
| maltose/maltodextrin transport system ATP-binding protein | 0.00098 | 36 |
| putative spermidine/putrescine transport system ATP-binding protein | 0.00095 | 29 |
| sorbitol/mannitol transport system ATP-binding protein | 0.00095 | 36 |
| lipoprotein-releasing system ATP-binding protein [EC:3.6.3.-] | 0.0009 | 32 |
| branched-chain amino acid transport system permease protein | 0.00088 | 30 |

**Supplementary Table S8.** Abundances of TIGRFAMs domains. Absolute counts and the relative abundances of the top 10 periphyton protein domains found in the TIGRFAMs database.

| **TIGRFAMs description** | **TIGRFAMs Accession** | **Relative**  **abundance** | **Absolute counts** |
| --- | --- | --- | --- |
| Type I secretion system ATPase | TIGR03375, TIGR01842, TIGR01846 | 0.02 | 2193 |
| Thiol reductant ABC exporter, cydd subunit | TIGR02857 | 0.01 | 816 |
| Phosphonate ABC transporter, ATP-binding protein | TIGR02315 | 0.01 | 810 |
| Daunorubicin resistance ABC transporter, ATP-binding protein | TIGR01188 | 0.01 | 781 |
| Cell division ATP-binding protein ftse | TIGR02673 | 0.01 | 780 |
| Sulfate ABC transporter, ATP-binding protein | TIGR00968 | 0.01 | 771 |
| ABC transporter, ATP-binding subunit, pqq-dependent alcohol dehydrogenase system | TIGR03864 | 0.01 | 763 |
| Thiol reductant ABC exporter, cydc subunit | TIGR02868 | 0.01 | 762 |
| Putative bacteriocin export ABC transporter, lactococcin 972 group | TIGR03608 | 0.01 | 760 |
| Putative 2-aminoethylphosphonate ABC transporter, ATP-binding protein | TIGR03265 | 0.01 | 751 |

Supplementary Table S9. Carbon fixation pathways in prokaryotes. Relative abundances of modules under the KEGG pathway Carbon fixation pathways in prokaryotes and absolute counts of key enzymes in this pathway.

| **Pathway module** | **Relative abundance** | **Absolute counts of**  **key enzymes** |
| --- | --- | --- |
| Reductive citrate cycle (Arnon-Buchanan cycle) [PATH:map01200 map00720] | 0.0131 | 37 |
| 3-Hydroxypropionate bi-cycle [PATH:map01200 map00720] | 0.0119 | 1 |
| Hydroxypropionate-hydroxybutylate cycle [PATH:map01200 map00720] | 0.0118 | 0 |
| Dicarboxylate-hydroxybutyrate cycle [PATH:map01200 map00720] | 0.0106 | 0 |
| Reductive pentose phosphate cycle (Calvin cycle) [PATH:map01200 map00710] | 0.007 | 44 |
| Reductive pentose phosphate cycle, ribulose-5P => glyceraldehyde-3P [PATH:map01200 map00710] | 0.0037 | 0 |

Supplementary Table S10. Nitrogen metabolism in periphyton. KEGG Orthology enzymes and pathway modules that perform nitrogen metabolism. Taxonomic affiliations are those from the NCBI taxonomy analysis results at order and class level for each enzyme belonging to the corresponding pathway module

| **KO Accession** | **Enzyme name** | **Pathway module** | **Order level taxonomy** | **Class level taxonomy** |
| --- | --- | --- | --- | --- |
| K00360 | nitrate reductase (NADH) [EC:1.7.1.1] | Assimilatory nitrate reduction, nitrate => ammonia [PATH:map00910] | Enterobacteriales; Alteromonadales | Gammaproteobacteria |
| K00366 | ferredoxin-nitrite reductase [EC:1.7.7.1] | Assimilatory nitrate reduction, nitrate => ammonia [PATH:map00910] | Pseudomonadales; Gammaproteobacteria; Betaproteobacteria; Rhizobiales; Campylobacterales; Rhodobacterales; Alphaproteobacteria; Sphingobacteriales | Gammaproteobacteria; Alphaproteobacteria; Proteobacteria; Epsilonproteobacteria; Sphingobacteria |
| K00372 | nitrate reductase catalytic subunit [EC:1.7.99.4] | Assimilatory nitrate reduction, nitrate => ammonia [PATH:map00910] | Betaproteobacteria; Flavobacteriales; Verrucomicrobiales; Sphingomonadales; Enterobacteriales; Myxococcales; Rhodobacterales; Nautiliales; Cytophagia; Alphaproteobacteria; Planctomycetales; Burkholderiales; Alteromonadales | Gammaproteobacteria; Betaproteobacteria; Flavobacteria; Bacteroidetes; Planctomycetacia; Epsilonproteobacteria; Deltaproteobacteria; Alphaproteobacteria; Proteobacteria; Verrucomicrobiae |
| K00370 | nitrate reductase 1, alpha subunit [EC:1.7.99.4] | Denitrification, nitrate => nitrogen [PATH:map00910] | Alphaproteobacteria; Rhodobacterales; Rhizobiales | Alphaproteobacteria; Proteobacteria |
| K00374 | nitrate reductase 1, gamma subunit [EC:1.7.99.4] | Denitrification, nitrate => nitrogen [PATH:map00910] | Alphaproteobacteria; Rhizobiales | Alphaproteobacteria; Proteobacteria |
| K00376 | nitrous-oxide reductase [EC:1.7.2.4] | Denitrification, nitrate => nitrogen [PATH:map00910] | Flavobacteriales | Flavobacteria |
| K02567 | periplasmic nitrate reductase NapA [EC:1.7.99.4] | Denitrification, nitrate => nitrogen [PATH:map00910] | Betaproteobacteria; Flavobacteriales; Cytophagia | Bacteroidetes; Flavobacteria; Proteobacteria |
| K04561 | nitric-oxide reductase, cytochrome b-containing subunit I [EC:1.7.99.7] | Denitrification, nitrate => nitrogen [PATH:map00910] | Betaproteobacteria; Rhodobacterales; Oceanospirillales; Methylophilales; Alphaproteobacteria | Gammaproteobacteria; Alphaproteobacteria; Betaproteobacteria; Proteobacteria |
| K00362 | nitrite reductase (NAD(P)H) large subunit [EC:1.7.1.4] | Dissimilatory nitrate reduction, nitrate => ammonia [PATH:map00910] | Gammaproteobacteria; Rhizobiales; Vibrionales; Rhodobacterales; Alphaproteobacteria; Burkholderiales; Alteromonadales | Gammaproteobacteria; Alphaproteobacteria; Betaproteobacteria; Proteobacteria |
| K00363 | nitrite reductase (NAD(P)H) small subunit [EC:1.7.1.4] | Dissimilatory nitrate reduction, nitrate => ammonia [PATH:map00910] | Alphaproteobacteria; Rhodobacterales | Alphaproteobacteria; Proteobacteria |
| K00370 | nitrate reductase 1, alpha subunit [EC:1.7.99.4] | Dissimilatory nitrate reduction, nitrate => ammonia [PATH:map00910] | Alphaproteobacteria; Rhodobacterales; Rhizobiales | Alphaproteobacteria; Proteobacteria |
| K00374 | nitrate reductase 1, gamma subunit [EC:1.7.99.4] | Dissimilatory nitrate reduction, nitrate => ammonia [PATH:map00910] | Alphaproteobacteria; Rhizobiales | Alphaproteobacteria; Proteobacteria |
| K02567 | periplasmic nitrate reductase NapA [EC:1.7.99.4] | Dissimilatory nitrate reduction, nitrate => ammonia [PATH:map00910] | Betaproteobacteria; Flavobacteriales; Cytophagia | Bacteroidetes; Flavobacteria; Proteobacteria |

Supplementary Table S11. Abundances of Gene Ontology terms. Relative abundances of the top 10 terms found in the three domains of the Gene Ontology database.

| **Molecular Function** | **Relative Abundance** | **Biological Process** | **Relative Abundance** | **Cellular Component** | **Relative Abundance** |
| --- | --- | --- | --- | --- | --- |
| ATP binding | 0.067 | Translation | 0.026 | Cytoplasm | 0.072 |
| DNA binding | 0.042 | Nucleosome assembly | 0.019 | Nucleus | 0.022 |
| Metal ion binding | 0.025 | Oxidation-reduction process | 0.015 | Nucleosome | 0.018 |
| Structural constituent of ribosome | 0.024 | Protein folding | 0.011 | Integral to membrane | 0.016 |
| GTP binding | 0.022 | Response to stress | 0.0087 | Ribosome | 0.014 |
| GTPase activity | 0.018 | Transport | 0.0086 | Plasma membrane | 0.013 |
| DNA-directed RNA polymerase activity | 0.018 | ATP synthesis coupled proton transport | 0.0063 | Chloroplast thylakoid membrane | 0.0085 |
| rRNA binding | 0.016 | one-carbon metabolic process | 0.0054 | Small ribosomal subunit | 0.0069 |
| Protein binding | 0.013 | DNA repair | 0.0043 | Proton-transporting ATP synthase complex, catalytic core F(1) | 0.0051 |
| Translation elongation factor activity | 0.0097 | Glycolysis | 0.0036 | Chloroplast | 0.0043 |

Supplementary Table S12. Methane metabolism in periphyton. KEGG Orthology enzymes and pathway modules that perform methane metabolism. Taxonomic affiliations are those from the NCBI taxonomy analysis results at order and class level for each enzyme belonging to the corresponding pathway module.

| **KO Accession** | **Enzyme name** | **Pathway module** | **Order level taxonomy** | **Class level taxonomy** |
| --- | --- | --- | --- | --- |
| K00925 | acetate kinase [EC:2.7.2.1] | Methanogenesis, acetate => methane [PATH:map01200 map00680] | Thermoanaerobacterales; Gammaproteobacteria; Bacteroidia; Flavobacteriales; Rhizobiales; Vibrionales; Myxococcales; Rhodobacterales; Alphaproteobacteria; Bacteroidales; Burkholderiales | Gammaproteobacteria; Betaproteobacteria; Flavobacteria; Bacteroidetes; Bacteroidia; Deltaproteobacteria;  Alphaproteobacteria; Clostridia; Proteobacteria |
| K00625 | phosphate acetyltransferase [EC:2.3.1.8] | Methanogenesis, acetate => methane [PATH:map01200 map00680] | Flavobacteria; Rhizobiales; Chloroflexales; Rhodobacterales; Chloroflexi class; Alphaproteobacteria | Bacteroidetes; Chloroflexi class; Alphaproteobacteria; Chloroflexi; Proteobacteria |
| K13788 | phosphate acetyltransferase [EC:2.3.1.8] | Methanogenesis, acetate => methane [PATH:map01200 map00680] | Flavobacteriales; Flavobacteria; Rhizobiales; Rhodobacterales; Alphaproteobacteria; Desulfobacterales; Desulfovibrionales | Deltaproteobacteria; Bacteroidetes; Alphaproteobacteria; Flavobacteria; Proteobacteria |
| K01895 | acetyl-CoA synthetase [EC:6.2.1.1] | Methanogenesis, acetate => methane [PATH:map01200 map00680] | Neisseriales; Betaproteobacteria; Actinopterygii; Bdellovibrionales; Actinobacteria class; Clostridiales; Saccharomycetales; Cytophagia; Cytophagales; Chlorophyceae; Bacillales; Actinomycetales; Acidobacteriales; Rhodospirillales; Amphibia;  Alteromonadales; Burkholderiales; Pseudomonadales; Gammaproteobacteria; Flavobacteriales; Rhizobiales; Anthozoa;  Planctomycetacia; Rhodobacterales; Mammalia; Echinoidea; Alphaproteobacteria; Naviculales; Desulfovibrionales;  Verrucomicrobiales; Flavobacteria; Syntrophobacterales; Myxococcales; Chromatiales; Thermales; Aves; Sphingobacteriales; Planctomycetales; Chlorobiales | Chlorobia; Actinobacteria class; Acidobacteria class; Betaproteobacteria; Cytophagia; Bacillariophyceae; Proteobacteria; Planctomycetes; Chordata; Deinococci; Clostridia; Verrucomicrobiae; Bacilli; Gammaproteobacteria; Bacteroidetes; Planctomycetacia; Chlorophyta; Cnidaria; Alphaproteobacteria; Flavobacteria; Sphingobacteria; Actinobacteria;  Saccharomycetes; Deltaproteobacteria; Echinodermata |
| K00194 | acetyl-CoA decarbonylase/synthase complex subunit delta | Methanogenesis, acetate => methane [PATH:map01200 map00680] | Syntrophobacterales; Desulfobacterales | Deltaproteobacteria |
| K03388 | heterodisulfide reductase subunit A [EC:1.8.98.1] | Methanogenesis, acetate => methane [PATH:map01200 map00680] | Desulfobacterales | Deltaproteobacteria |
| K00320 | coenzyme F420-dependent N5,N10-methenyltetrahydromethanopterin reductase [EC:1.5.99.11] | Methanogenesis, CO2 => methane [PATH:map01200 map00680] | Actinomycetales; Rubrobacterales; Actinobacteria class | Actinobacteria class; Actinobacteria |
| K03388 | heterodisulfide reductase subunit A [EC:1.8.98.1] | Methanogenesis, CO2 => methane [PATH:map01200 map00680] | Desulfobacterales | Deltaproteobacteria |
| K03388 | heterodisulfide reductase subunit A [EC:1.8.98.1] | Methanogenesis, methanol => methane [PATH:map01200 map00680] | Desulfobacterales | Deltaproteobacteria |
| K14083 | trimethylamine methyltransferase [EC:2.1.1.-] | Methanogenesis, methylamine/dimethylamine/trimethylamine => methane [PATH:map01200 map00680] | Flavobacteriales; Rhizobiales; Methanomicrobia; Clostridiales; Rhodobacterales; Alphaproteobacteria; Desulfobacterales; Methanosarcinales | Flavobacteria; Methanomicrobia; Euryarchaeota; Deltaproteobacteria; Alphaproteobacteria; Clostridia; Proteobacteria |
| K03388 | heterodisulfide reductase subunit A [EC:1.8.98.1] | Methanogenesis, methylamine/dimethylamine/trimethylamine => methane [PATH:map01200 map00680] | Desulfobacterales | Deltaproteobacteria |

**Supplementary Table S13.** Abbreviations in Figure 3. Full names of the abbreviations of biochemical functions shown in Figure 3.

| **Abbreviation** | **Full name** |
| --- | --- |
| Met. | Metabolism |
| Unclssfd | Unclassified |
| T&C | Transport and Catabolism |
| CC | Cell Communication |
| CM | Cell Motility |
| CG&D | Cell Growth and Death |
| Signal Tra. | Signal Transduction |
| Membrane Tra. | Membrane Transport |
| SM&I | Signaling Molecules and Interaction |
| Trnscrptn | Transcription |
| Trnsltn | Translation |
| FS&D | Folding Sorting and Degradation |
| Rep. & Rep. | Replication and Repair |
| ND | Neurodegenerative Diseases |
| ID | Infectious Diseases |
| NM | Nucleotide Metabolism |
| GB&M | Glycan Biosynthesis and Metabolism |
| LM | Lipid Metabolism |
| MoC&V | Metabolism of Cofactors and Vitamins |
| XB&M | Xenobiotics Biodegradation and Metabolism |
| Enzyme Fam. | Enzyme Families |
| Car. Met. | Carbohydrate Metabolism |
| D | Development |
| DS | Digestive System |
| ES | Endocrine System |
| IS | Immune System |
| NS | Nervous System |
| CP&S | Cellular Processes and Signaling |
| PC | Poorly Characterized |
| Reg. of. act. cyt. | Regulation of actin cytoskeleton |
| Cytoskeleton pro. | Cytoskeleton proteins |
| Bacterial mot. pro. | Bacterial motility proteins |
| Bacterial che. | Bacterial chemotaxis |
| Two-component sys. | Two-component system |
| Bacterial sec. sys. | Bacterial secretion system |
| Transcription fac. | Transcription factors |
| Aminoacyl-tRNA bio. | Aminoacyl-tRNA biosynthesis |
| Cha. & fol. cat. | Chaperones and folding catalysts |
| DNA rep. & rec. | DNA repair and recombination proteins pro. |
| DNA rep. pro. | DNA replication proteins |
| Systemic lup. ery. | Systemic lupus erythematosus |
| Geraniol deg. | Geraniol degradation |
| Terpenoid bac. bio. | Terpenoid backbone biosynthesis |
| Tetracycline bio. | Tetracycline biosynthesis |
| Pol. sug. uni. bio. | Polyketide sugar unit biosynthesis |
| Peptidoglycan bio. | Peptidoglycan biosynthesis |
| Lip. bio. pro. | Lipopolysaccharide biosynthesis proteins |
| Cfpip | Carbon fixation pathways in prokaryotes |
| Oxidative pho. | Oxidative phosphorylation |
| Cfipo | Carbon fixation in photosynthetic organisms |
| One car. pool by fol. | One carbon pool by folate |
| Pan. & CoA. bio. | Pantothenate and CoA biosynthesis |
| Por. & chl. met. | Porphyrin and chlorophyll metabolism |
| Nic. & nic. met. | Nicotinate and nicotinamide metabolism |
| Ubi. & oth. ter. bio. | Ubiquinone and other terpenoid-quinone biosynthesis |
| Benzoate deg. | Benzoate degradation |
| Chl. & chl. deg. | Chloroalkane and chloroalkene degradation |
| Drug met. - oth. enz. | Drug metabolism - other enzymes |
| Val. leu. & iso. deg. | Valine leucine and isoleucine degradation |
| Arginine & pro. met. | Arginine and proline metabolism |
| Ala. asp. & glu. met. | Alanine aspartate and glutamate metabolism |
| Gly. ser. & thr. met. | Glycine serine and threonine metabolism |
| Val. leu. & iso. bio. | Valine leucine and isoleucine biosynthesis |
| Amino acid rel. enz. | Amino acid related enzymes |
| Gly. & dic. met. | Glyoxylate and dicarboxylate metabolism |
| Glycolysis / Glu. | Glycolysis / Gluconeogenesis |
| Cit. cyc. (TC. cyc. | Citrate cycle (TCA cycle) |
| As&nsm | Amino sugar and nucleotide sugar metabolism |
| Starch & suc. met. | Starch and sucrose metabolism |
| Plant-pathogen int. | Plant-pathogen interaction |
| Cell mot. & sec. | Cell motility and secretion |
| Other ion. tra. | Other ion-coupled transporters |
| Signal tra. mec. | Signal transduction mechanisms |

## Supplementary Figures
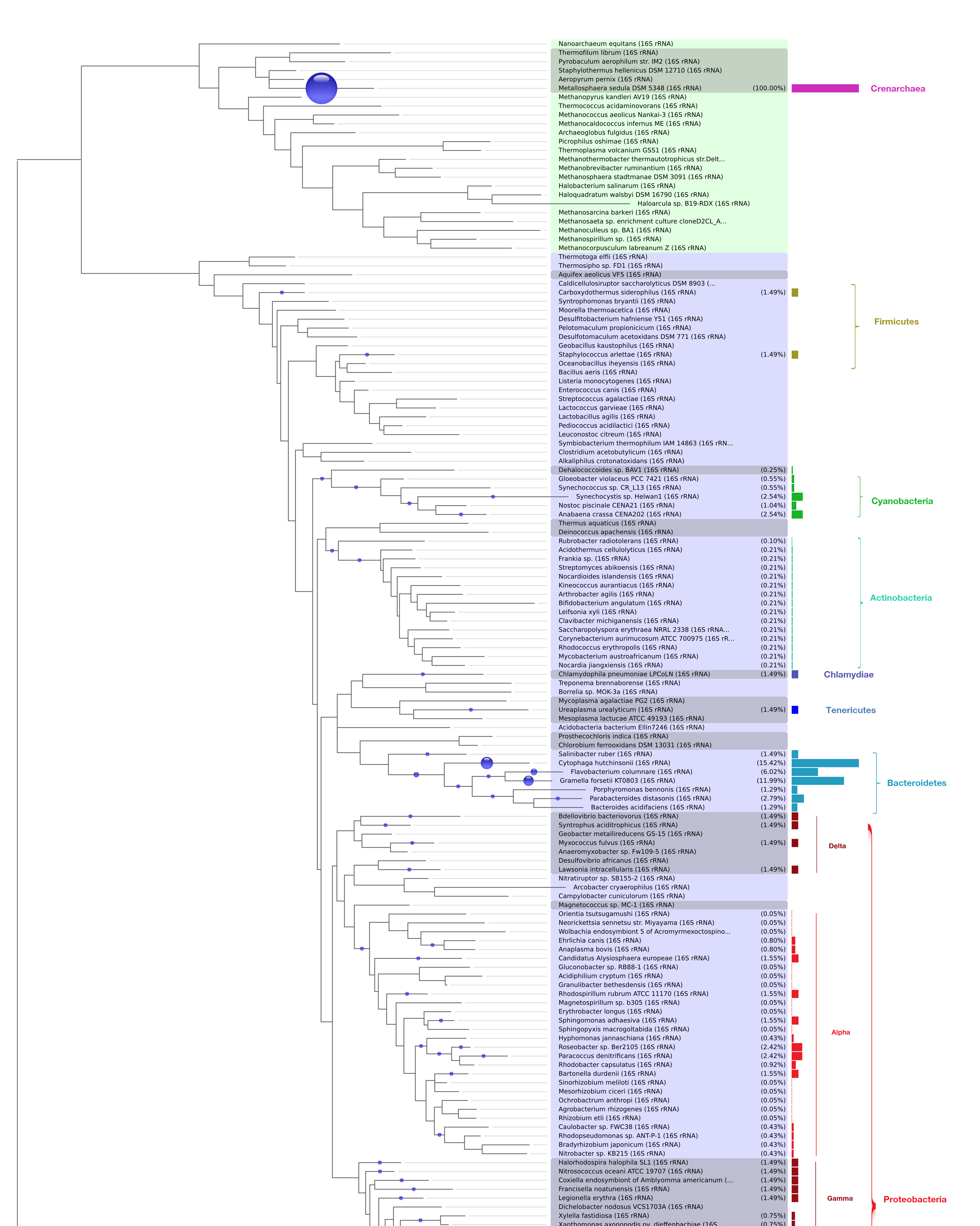


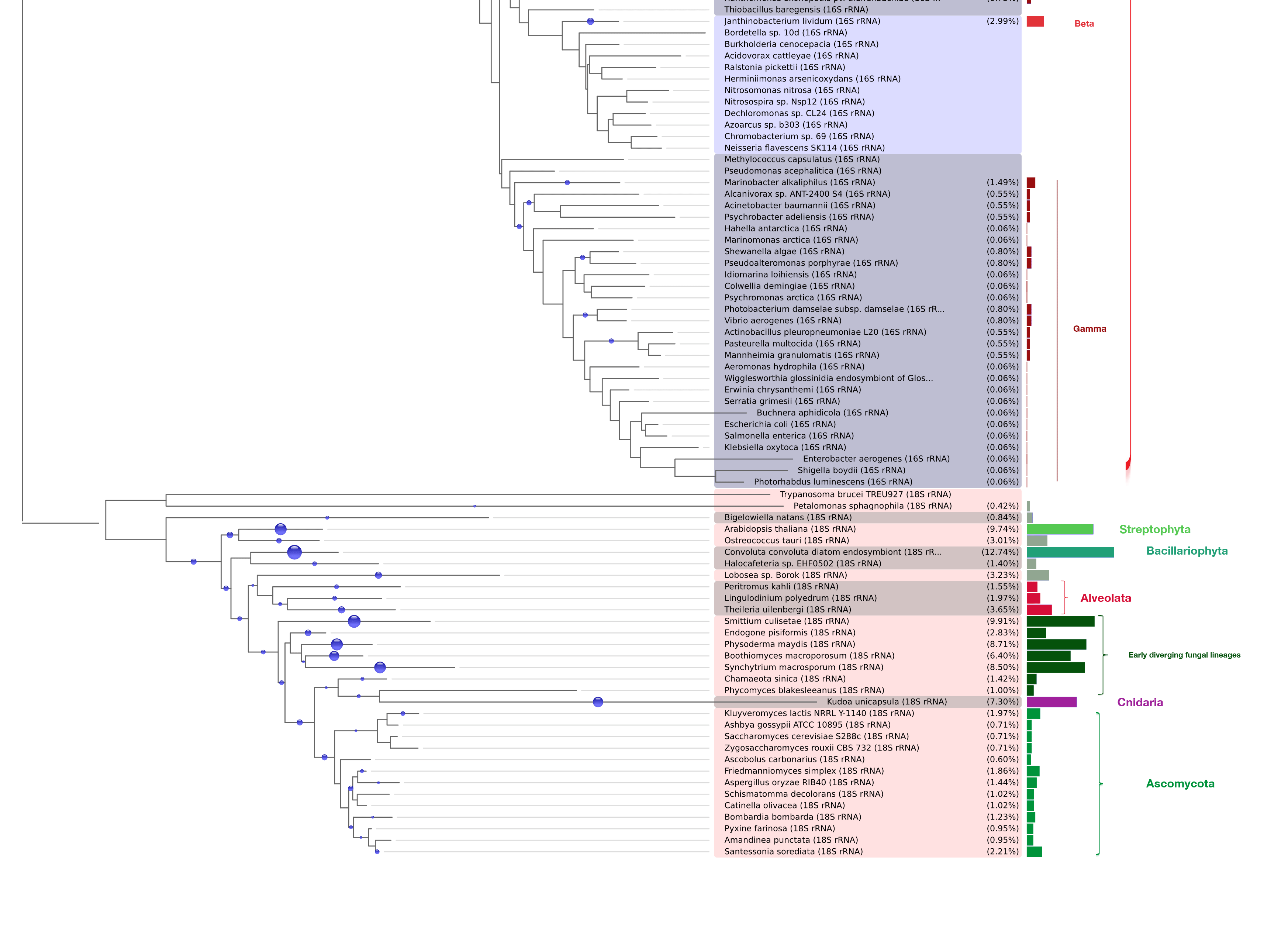


**Supplementary Figure S1.** Phylogenetic tree for periphyton. Phylogenetic tree inferred using SSU rRNA sequences extracted from periphyton metagenomes in MLTreeMap. Species are highlighted by their domains: Archaea (green), Bacteria (blue), Eukaryotes (pink). Circles correspond to likelihood of the placement of sequence to the corresponding node in the tree. Bars represent summation of the likelihood values for the corresponding species in the leaf node.

**
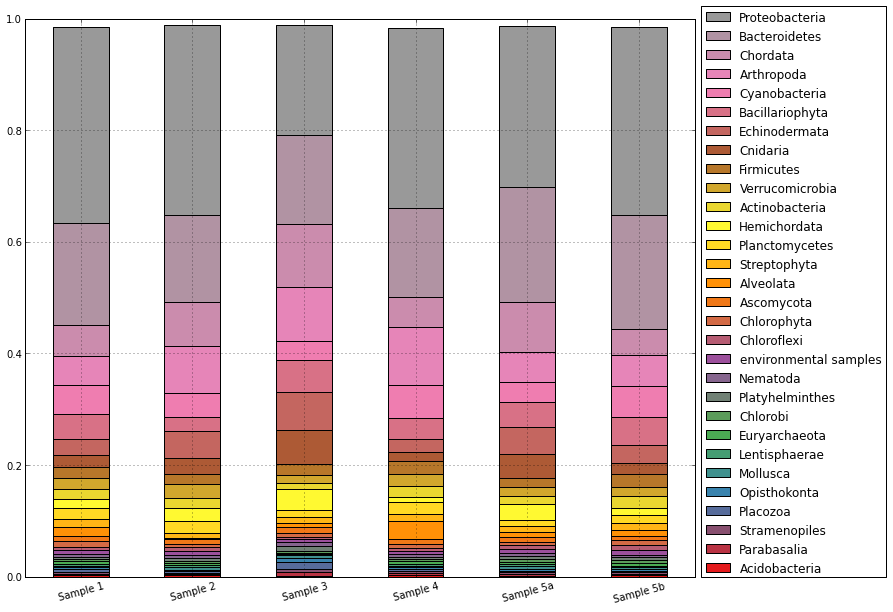
**

**Supplementary Figure S2.** Relative abundances of phyla in each sample**,** calculated according to the blastx matches to the NCBI nr database.

**
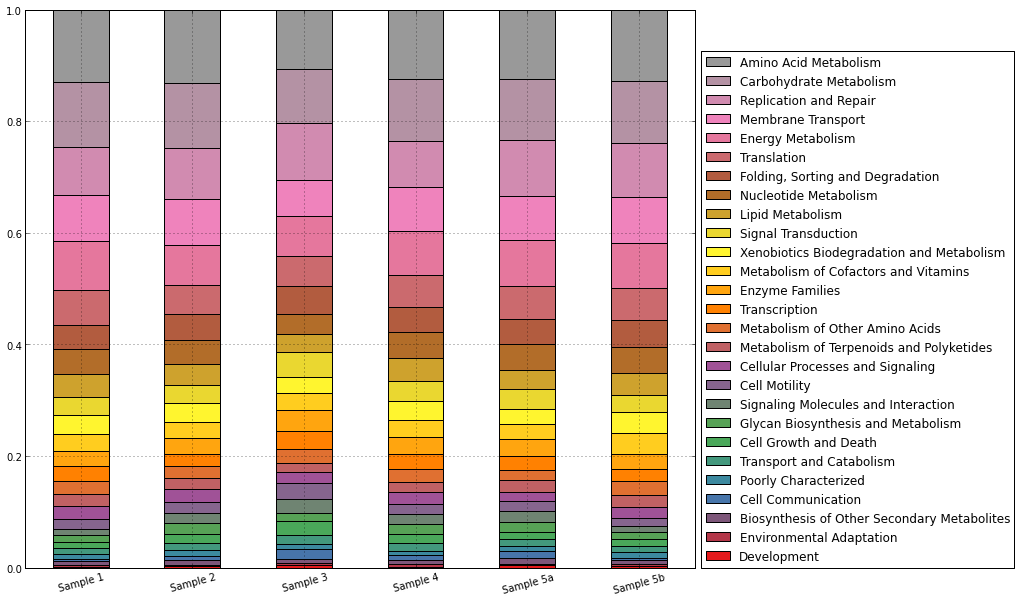
**

**Supplementary Figure S3.** Relative abundances of KEGG Orthology annotations in each sample**,** calculated according to the blastp matches to the second hierarchy level of the KEGG Brite database.


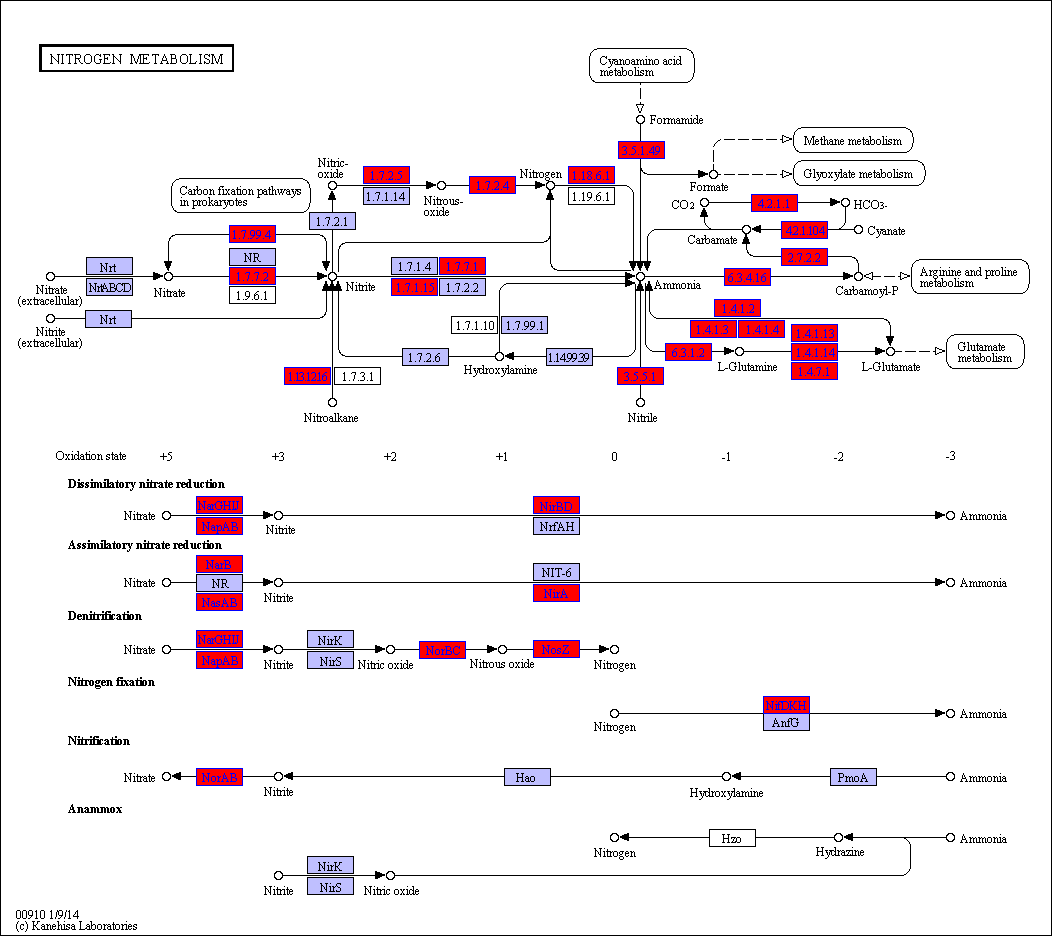


**Supplementary Figure S4.** Nitrogen metabolism in periphyton. Metabolic pathway map of the nitrogen metabolism functions found in periphyton. Red boxes represent the enzymes found in the periphyton metagenome.


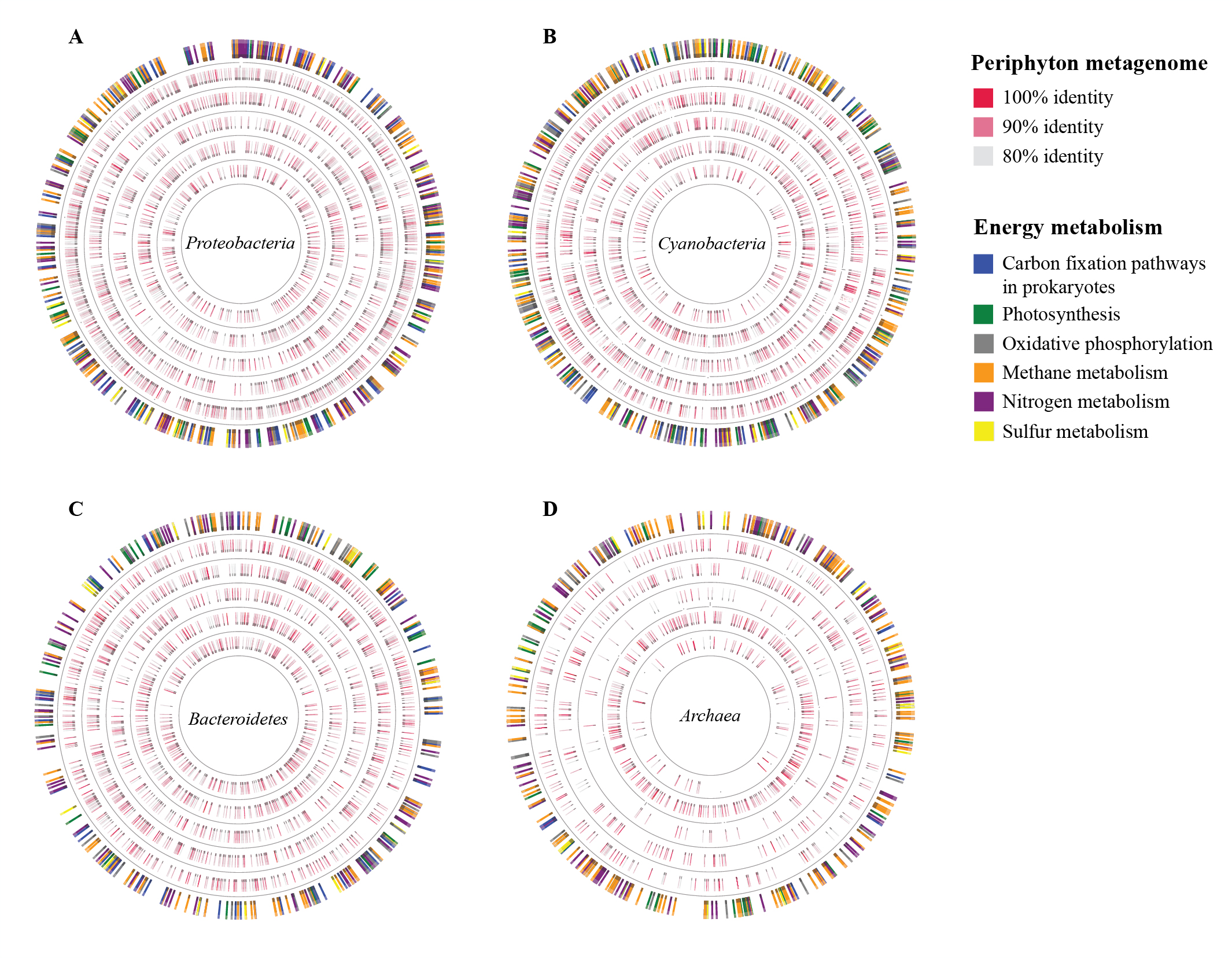


**Supplementary Figure S5. Sequence mapping of the periphyton metagenome to reference genomes.** Sequence mapping results of the periphyton metagenome to the reference genomes from the phyla a. *Proteobacteria*, b. *Cyanobacteria*, c. *Bacteroidetes* and d. the kingdom archaea. The outer-most circles with the colored bars, represent the loci taking role in energy metabolism within the genome of the outer-most circular genome. Species strains selected for the genome mapping for each phyla (inner to outer circles) are as follows: a. *Bdellovibrio bacteriovorus* strain 109J, *Halorhodospira halophila* SL1, *Janthinobacterium sp*. Marseille, *Paracoccus denitrificans* PD1222, *Roseobacter denitrificans* OCh 114, b. *Dehalococcoides sp*. BAV1, *Gloeobacter violaceus* PCC 7421, *Synechocystis sp*. PCC 6803, *Nostoc punctiforme* PCC 73102, *Anabaena variabilis* ATCC 29413, c. *Parabacteroides distasonis* ATCC 8503, *Cytophaga hutchinsonii* ATCC 33406, *Bacteroides fragilis* NCTC 9343, *Flavobacterium columnare* ATCC 49512, *Gramella forsetii* KT0803, and d. *Metallosphaera sedula* DSM 5348, *Methanocaldococcus jannaschii* DSM 2661, *Methanopyrus kandleri* AV19,*Halobacterium salinarum* R1, *Methanosarcina barkeri* str. Fusaro.

**References**

Barken, K.B., Pamp, S.J., Yang, L., Gjermansen, M., Bertrand, J.J., Klausen, M., Givskov, M., Whitchurch, C.B., Engel, J.N., and Tolker-Nielsen, T. (2008). Roles of type IV pili, flagellum-mediated motility and extracellular DNA in the formation of mature multicellular structures in Pseudomonas aeruginosa biofilms. *Environmental Microbiology* 10**,** 2331-2343. doi: 10.1111/j.1462-2920.2008.01658.x.

Borlee, B.R., Goldman, A.D., Murakami, K., Samudrala, R., Wozniak, D.J., and Parsek, M.R. (2010). Pseudomonas aeruginosa uses a cyclic-di-GMP-regulated adhesin to reinforce the biofilm extracellular matrix. *Molecular Microbiology* 75**,** 827-842. doi: 10.1111/j.1365-2958.2009.06991.x.

Canfield, D.E., Thamdrup, B., and Kristiensen, E. (2005). "Microbial Mats," in *Aquatic Geomicrobiology*. Elsevier Academic Press), 466-480.

Flemming, H.C., and Wingender, J. (2010). The biofilm matrix. *Nature Reviews Microbiology* 8**,** 623-633. doi: 10.1038/nrmicro2415.

Frolund, B., Palmgren, R., Keiding, K., and Nielsen, P.H. (1996). Extraction of extracellular polymers from activated sludge using a cation exchange resin. *Water Research* 30**,** 1749-1758. doi: 10.1016/0043-1354(95)00323-1.

Gallaher, T.K., Wu, S., Webster, P., and Aguilera, R. (2006). Identification of biofilm proteins in non-typeable Haemophilus Influenzae. *Bmc Microbiology* 6**,** 9. doi: 10.1186/1471-2180-6-65.

Givskov, M., Denys, R., Manefield, M., Gram, L., Maximilien, R., Eberl, L., Molin, S., Steinberg, P.D., and Kjelleberg, S. (1996). Eukaryotic interference with homoserine lactone-mediated prokaryotic signaling. *Journal of Bacteriology* 178**,** 6618-6622.

Hahn, M.W., and Hofle, M.G. (1999). Flagellate predation on a bacterial model community: Interplay of size-selective grazing, specific bacterial cell size, and bacterial community composition. *Applied and Environmental Microbiology* 65**,** 4863-4872.

Krohn-Molt, I., Wemheuer, B., Alawi, M., Poehlein, A., Gullert, S., Schmeisser, C., Pommerening-Roser, A., Grundhoff, A., Daniel, R., Hanelt, D., and Streit, W.R. (2013). Metagenome Survey of a Multispecies and Alga-Associated Biofilm Revealed Key Elements of Bacterial-Algal Interactions in Photobioreactors. *Applied and Environmental Microbiology* 79**,** 6196-6206. doi: 10.1128/aem.01641-13.

Pang, J.M., Layre, E., Sweet, L., Sherrid, A., Moody, D.B., Ojha, A., and Sherman, D.R. (2012). The Polyketide Pks1 Contributes to Biofilm Formation in Mycobacterium tuberculosis. *Journal of Bacteriology* 194**,** 715-721. doi: 10.1128/jb.06304-11.

Pasmore, M., and Costerton, J.W. (2003). Biofilms, bacterial signaling, and their ties to marine biology. *Journal of Industrial Microbiology & Biotechnology* 30**,** 407-413. doi: 10.1007/s10295-003-0069-6.

Sorensen, S.J., Bailey, M., Hansen, L.H., Kroer, N., and Wuertz, S. (2005). Studying plasmid horizontal transfer in situ: A critical review. *Nature Reviews Microbiology* 3**,** 700-710. doi: 10.1038/nrmicro1232.
